# Supplementary material for: Modeling genotypes in their microenvironment to predict single- and multi-cellular behavior
Source: Gigascience. 2019 Jan 31;8(3):giz010. doi: 10.1093/gigascience/giz010 (PMC6423375; doi:10.1093/gigascience/giz010)
Supplement: Supplemental Files [file giz010_supplemental_files.zip › Protocol.docx]

microC: A 3D virtual microenvironment for perturbation biology

**Introduction**

microC is a multiscale virtual microenvironment for perturbation biology. It enables experiments that link genotype to phenotype taking into account the surrounding microenvironment. microC has a modular architecture that enables a wide variety of experiment. Furthermore, it offers easy access to advanced computational modelling and supercomputing resources to the wider scientific community.

microC combines agent-based and gene network modelling and uses partial differential equations to simulate interactions among cells.

The tool itself does not require any installation on the user’s machine, as it can be accessed using a web browser. Experimental results and data are also available via a web interface. This protocol describes the process of preparing and submit an experiment with microC, and interpreting the simulation results.

**Procedure**

**Step 1: Accessing microC**

microC is available via <microc.org>. During the first visit this step might take several seconds. This will not be the case in future visits.

**Step 2: Preparing an experiment**


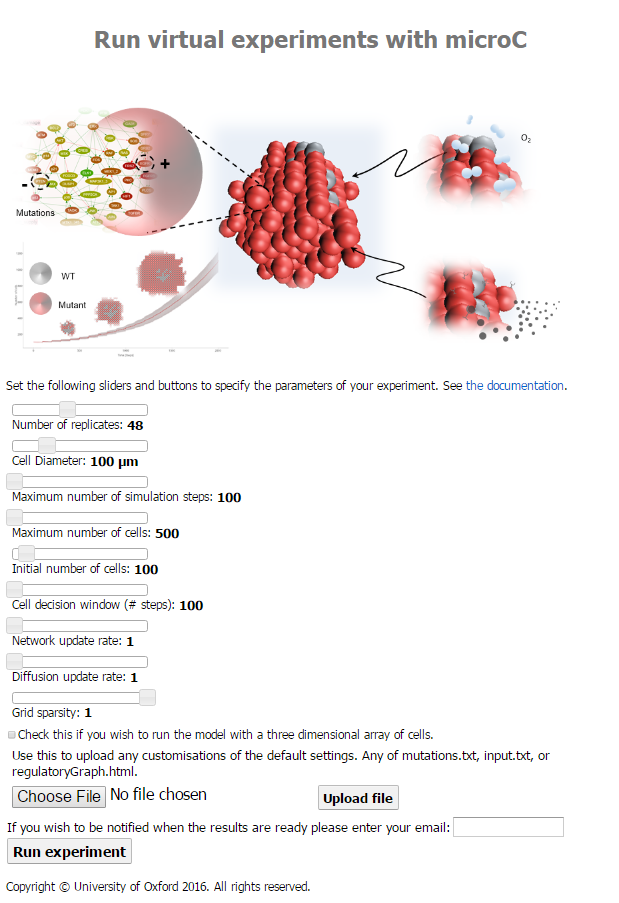


**Figure 1. microC main Interface**. Sliders are used to setup the numerical parameters, whereas the “Choose File” button may be used to upload local files that define the experiment. The check box enables switching between 2D/3D simulating environment. Finally, the “Run experiment” button submits the experiment.

Experiments are specified via the microC main web page (Figure 1). The user may set a number of numerical parameters via sliders (Table 1). Specifications of the gene network, mutations, and parameters that control diffusion can also be uploaded from the same page (Table 2 and Figure 2). microC modules are enabled through the specification files. For example setting parameters for *Oxygen* or any output node within diffusion-parameters.txt, will automatically enable a diffusion module for this substance.

**Table 1**. Parameters in microC interface.

| **Parameter** | **Parameter**  **(backend name)** | **Range**  **min – max, (increment)** | **Explanation** |
| --- | --- | --- | --- |
| Number of replicates | the-number-of-replicates | 16* – 92 (16) | Number of times this experiment should be repeated |
| Cell Diameter | the-cell-size | 10 – 300 (1) | Diameter of cell in μm (smaller cell-size simulations take longer to run) |
| Maximum number of simulation steps | the-maximum-number-of-ticks | 100* – 10000 (100) | Temporal steps this experiment will last |
| Maximum number of cells | the-maximum-cell-count | 100 – 4000 (1) | Maximum number of cells for each experimental replicate (large values may cause the simulation to run too slowly) |
| Initial number of cells | the-initial-cell-count | 1 – 1000 (1) | Number of cells at the start of each experimental replicate |
| Cell decision window | the-intercellular-step | 1 – 100000 (10) | Number of steps to take before cells actions (relative to microC time unit for simulation) |
| Network update rate | the-intracellular-step | 1 – 10000 (1) | Rate at which the gene networks are simulated (relative to microC time unit for simulation) |
| Diffusion update rate | the-diffusion-step | 1 – 1000 (1) | Rate at which diffusion is simulated (relative to microC time unit for simulation) |
| Grid sparsity | the-sparse-grid | 0 – 1 (1) | Sparsity of the grid in which diffusion is simulated (setting Grid sparsity to 0 will slow down the simulation) |

* Experiments with 16 replicates and less than 200 simulation steps (maximum number of simulation steps) are submitted in ARC’s developers’ queue. This options is meant for testing very short experiments to determine the final setup.

**Table 2**. Specification files microC experiments.

| **File name*** | **Function** | **Default option** |
| --- | --- | --- |
| mutations.txt | Specifies the mutation profiles of all clones in the experiment. | Wild type cells. Only one clone with no mutations. |
| input-parameters.txt | Specifies the response of receptor nodes of the network to environmental stimuli. | Initial values for all receptor nodes (except for oxygen) are 0 and thresholds values are 0.5. For the oxygen receptor the initial concentrations is 0.28mM and the threshold value 0.02mM. |
| associations.txt | Associates output and receptors nodes that enables cell-cell and cell-microenvironment interaction. | Empty. No associations between output and receptor nodes. |
| diffusion-parameters.txt | Specifies the parameter that determine diffusion of substances in the simulation. | Empty. No diffusible parameters. |
| regulatorygraph.html | Specifies the network that is simulated within the cells. | MAPK network (Figure 3) |

* microC only considers files with the specific names. Files with different names, are ignored and replaced by the default options.

| 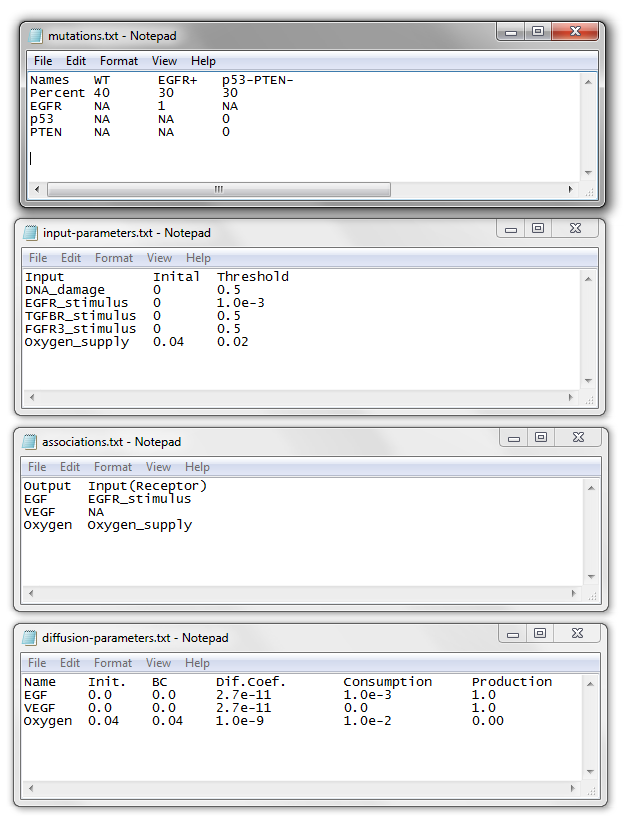 | 1. Names of clones  2. Percentage of clones in population (adds to 100)  3-5. Mutations for clonal subpopulatation (Gene name as in network, **1**: activating mutation/overexpression, **0**: loss of function mutation, **NA**: not specified). |
| --- | --- |
| 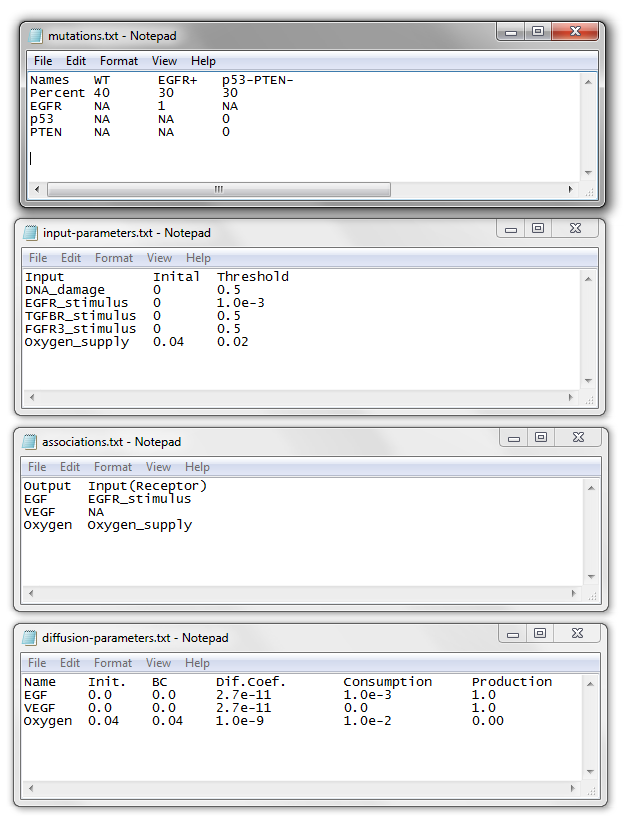 | 1. Column names  2-5. Receptor nodes properties (name as in network, initial value, activation threshold) |
| 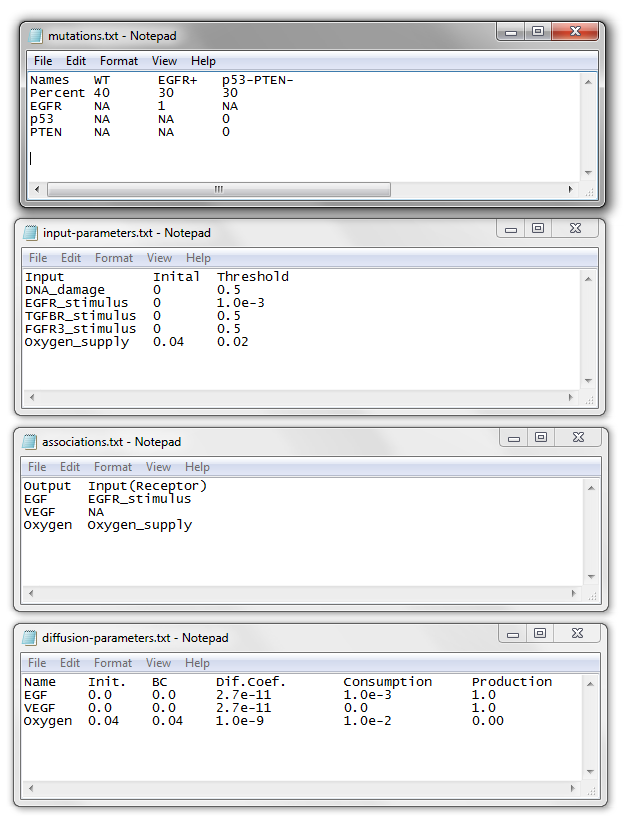 | 1. Column names  2-4. Association between output node and receptor (input) node (name of output node, name of receptor node) |
| 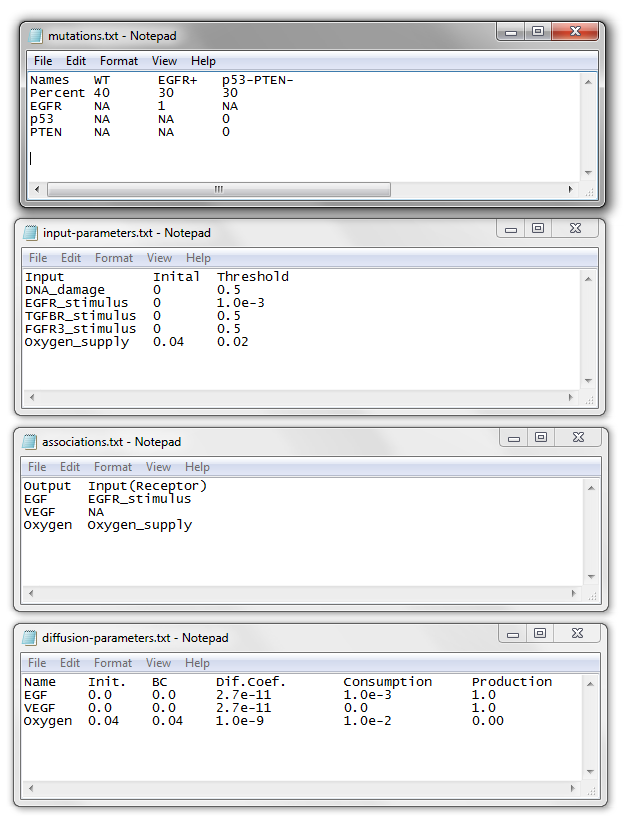 | 1. Column names  2-4. Diffusion parameters for substances (Initial concentration, boundary concentration, diffusion coefficient, consumption rate, production rate) |

**Figure 2.** Examples of files used by microC and their underlying structure. All files are tab seperated.


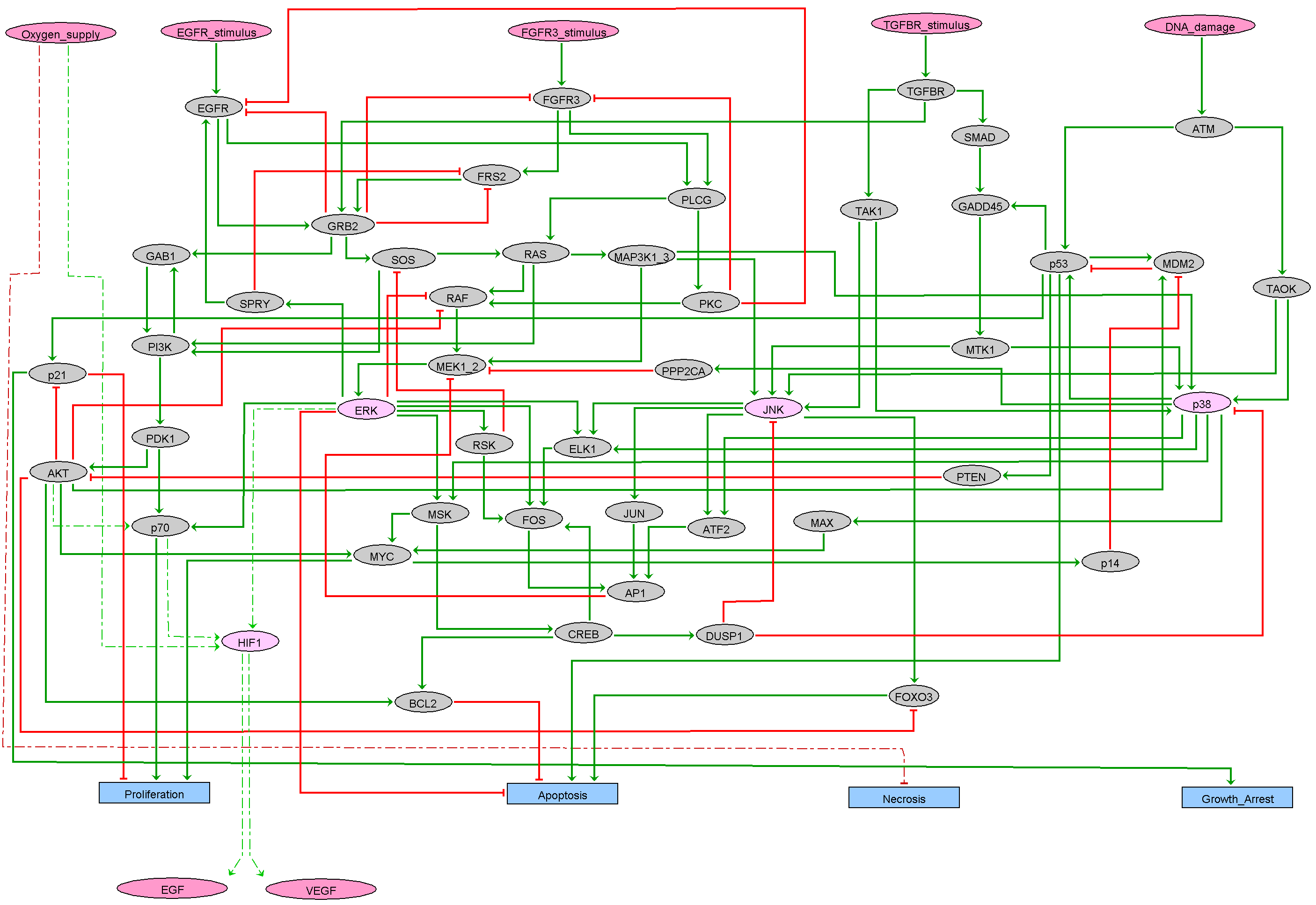


**Figure 3.** MAPK network used by default in microC. Solid lines represent the adopted MAPK network [Grieco et al., 2013. PLoS Comput Biol 9(10)], whereas the dashed lines represent extensions of the network related to the hypoxia signalling module. Receptor (input), output, and cell-fate nodes are optional for microC simulations. Cell-fate nodes named *Proliferation*, *Apoptosis*, *Necrosis*, and *Growth_Arrest*are supported by appropriate cell actions when activated.

**Step 3: Submitting a job**

Upon submission of the experiment (“Run Experiment” button, Figure 1) the user receives a link (Figure 4) that provides information about the status of the submission and the log file, where potential errrors are shown. Once the experiment is finished the same link will contain the results (that gradually become available in batches 16 replicates). Optionally, the user will be notified by email.


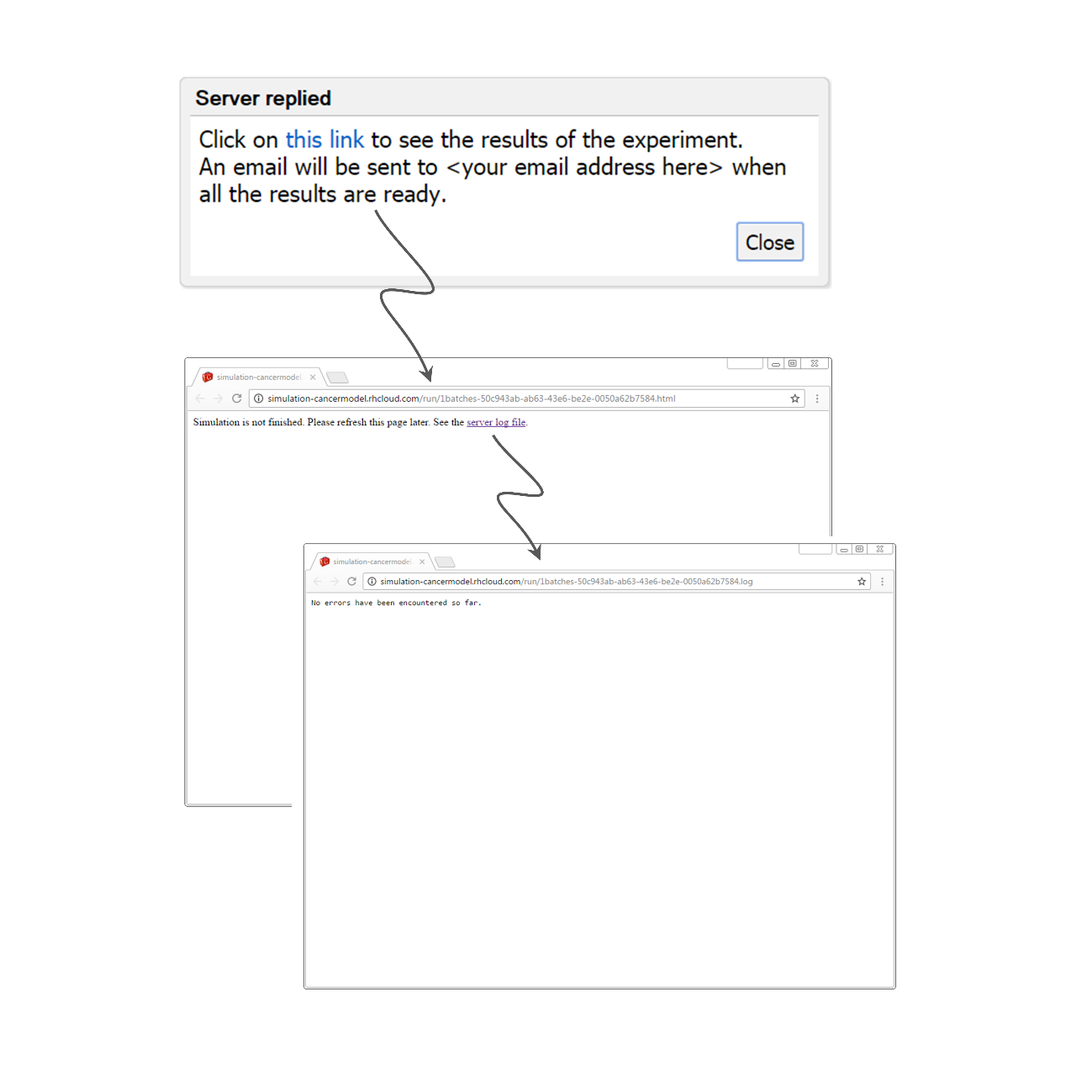


**Figure 4.** Upon sumission the user receives a confirmition and a link that will contain the results when the simulations will be finished.

**Step 4: Evaluating the results**

The results page displays data across all replicates and permits the viewer to inspect any of the replicates in detail. An animation of all replicates is displayed in the initial page along with averaged and detailed data for each of the experimental replicates (Figure 5).

| 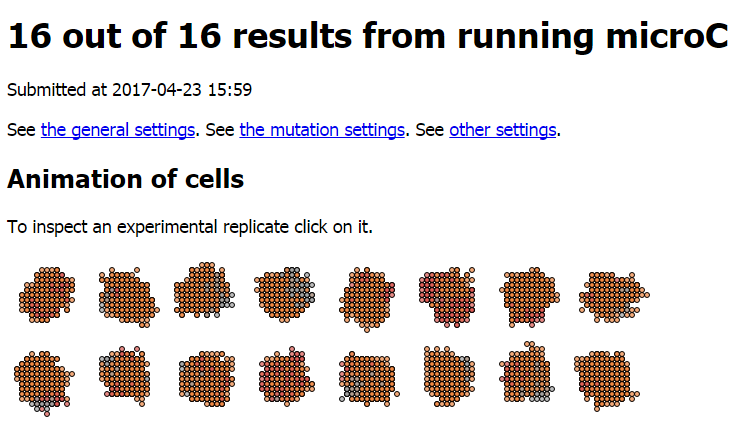 | Overview of the experimental replicates. Results display as animations. Also, the submission date and links to setting (see below) are displayed. |
| --- | --- |
| 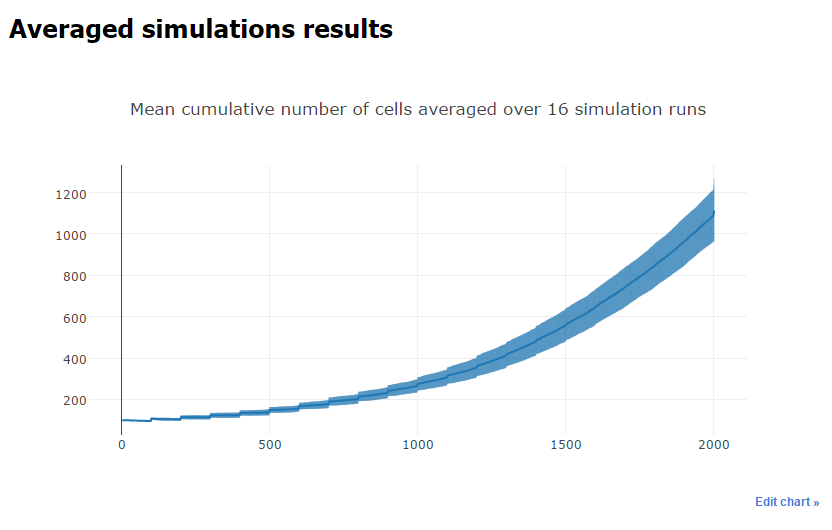 | Averaged cumulative data over all experimental replicates (error bars represent standard deviation): Number of cells, proliferation, apoptosis, growth arrest, and necrosis events. |
| … |  |
| 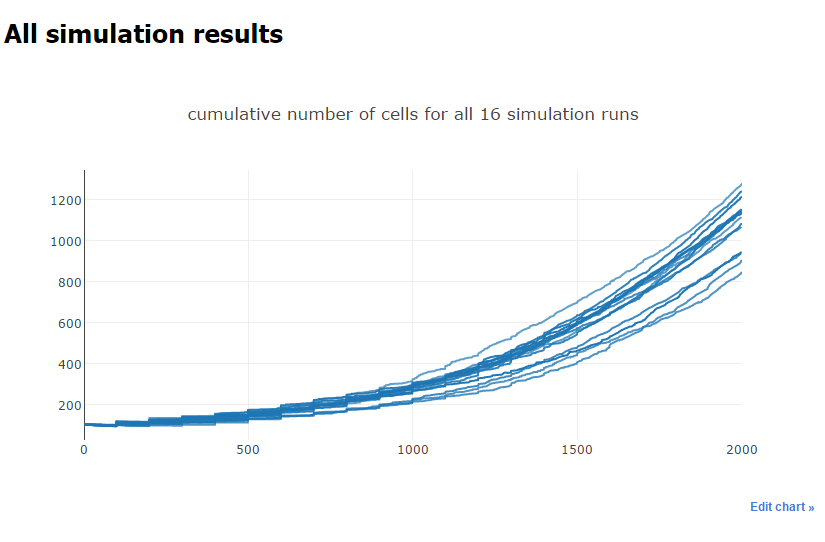 | Cumulative simulation data for each experimental replicate: Number of cells, proliferation, apoptosis, growth arrest, and necrosis events. |
| … |  |
| 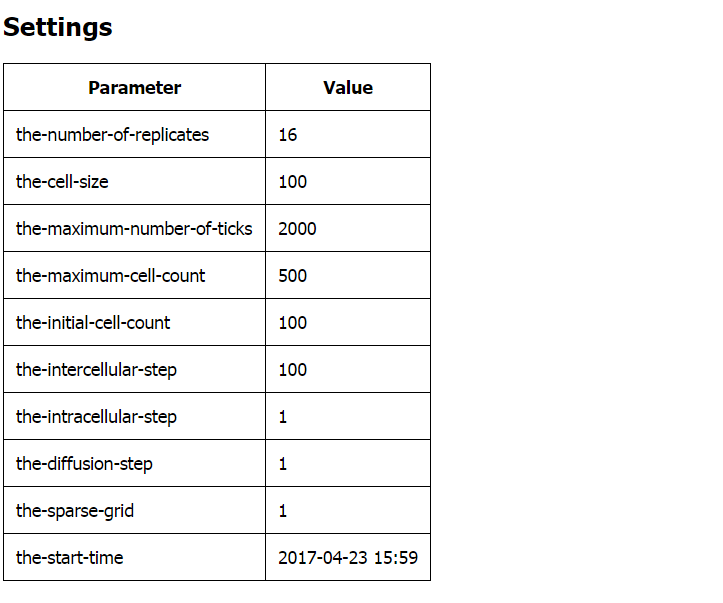 | The numerical parameters used for the particular experiment (set by the sliders in main web interface, Figure 1). These can be used to replicate the experiment or explore variants. |
| 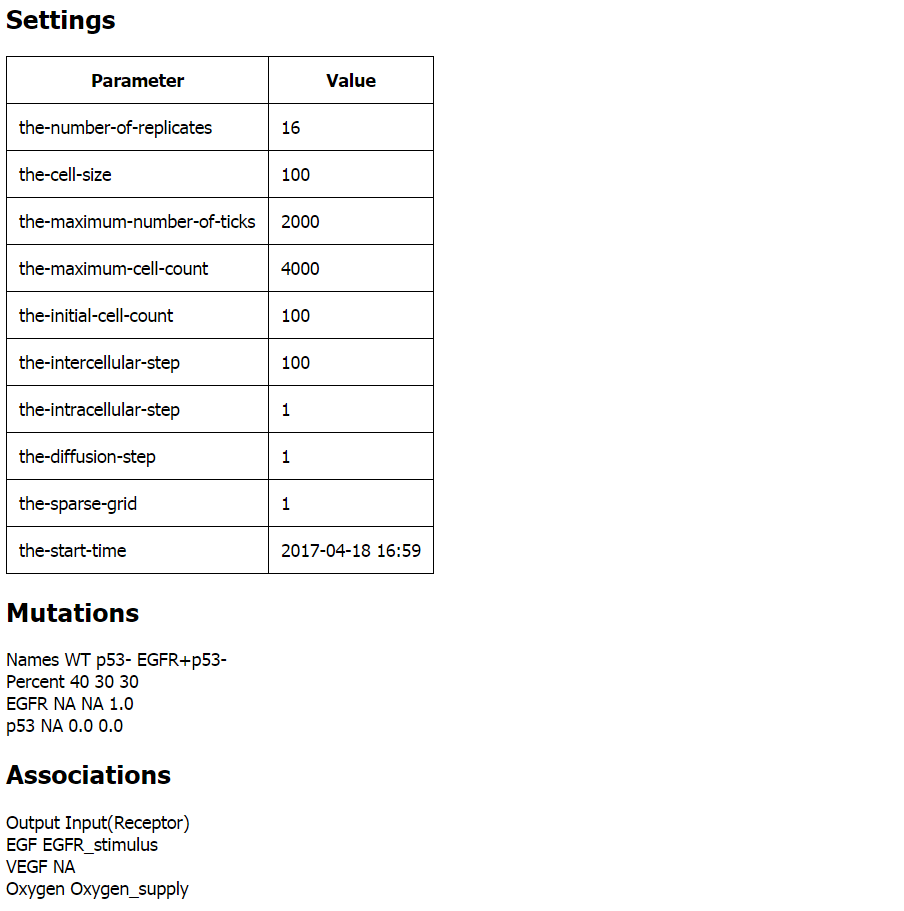  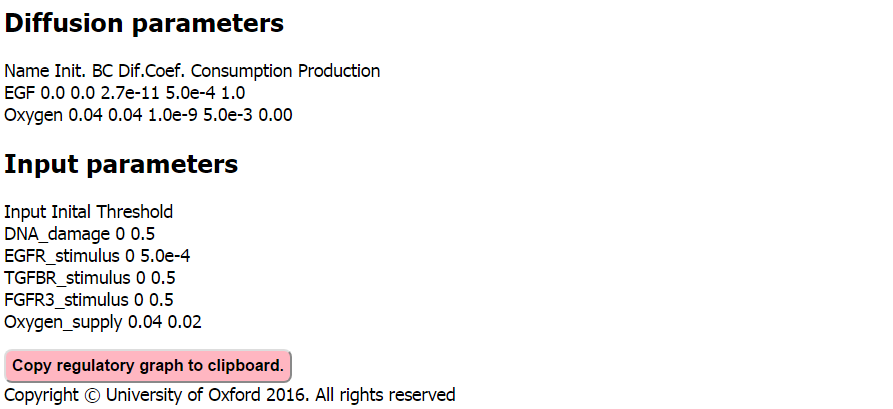 | Content of the microC files used for the particular experiment. The regulatory graph may be copied by pressing the “Copy regulatory graph function to clipboard”. |

**Figure 5.** Overview of microC experimental results.

Clicking on one of the experimental replicates the user may follow via an animation all events that happened during the experiment for the particular replicate (Figure 6).

| 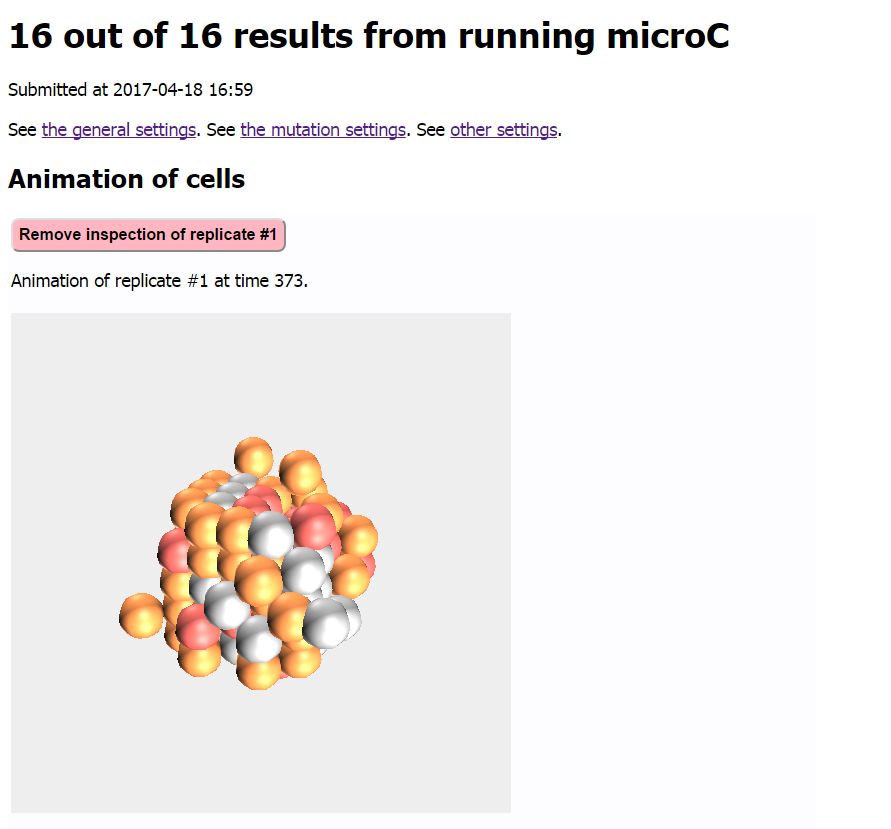 | Animation of the cells in two or three dimensions (as determined by the experimenter). The viewer sees all changes that took place during the simulations (proliferations and cell deaths). Different clone types are displayed with different colours, reflecting the mutation profile. The 3D visualisation supports rotation of the cell model for viewing at any angle. The replicate inspection may be terminated by pressing the “Remove inspection of replicate” button. |
| --- | --- |
| 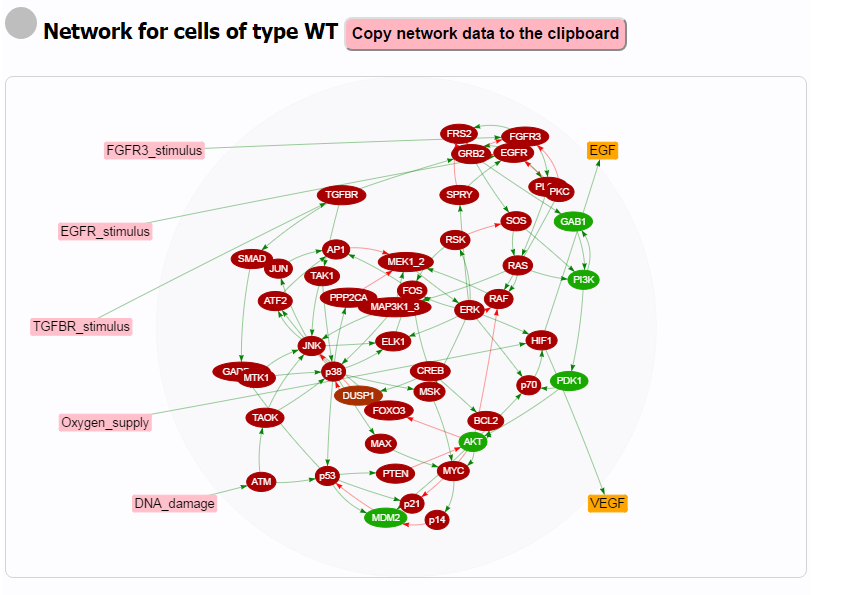  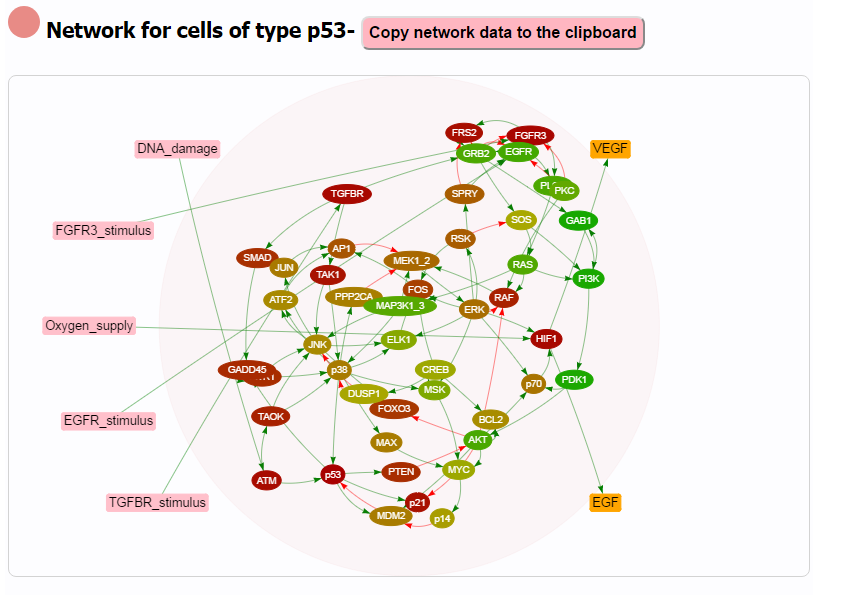 | The gene network of each clone is displayed along with the cell model animation. The gene colour represents the activation status of that gene across the clonal population. Hovering the mouse over one of the genes displays the exact percentage of activations and the average number of times the status changed during the simulations. The activation data for all genes throughout the simulation may be copied by pressing the “Copy network to the clipboard” button. |
| … |  |
| 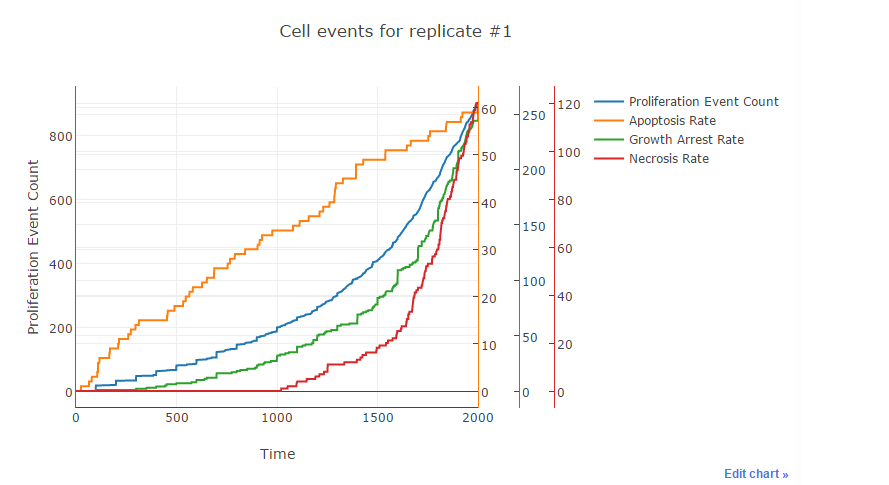 | Cumulative simulation data for the specific experimental replicate: proliferation, apoptosis, growth arrest, and necrosis events. |
|  |  |

**Figure 6.** Overview of microC experimental results (replicate inspection).

The graphs displayed by microC use the Plotly visualisation library (<https://plot.ly/>). This library supports interactions such as zooming into a portion of the history and reading off exact data values by hovering the mouse over a data point on a graph (Figure 7). It also supports exporting the underlying data in spreadsheet format.

**
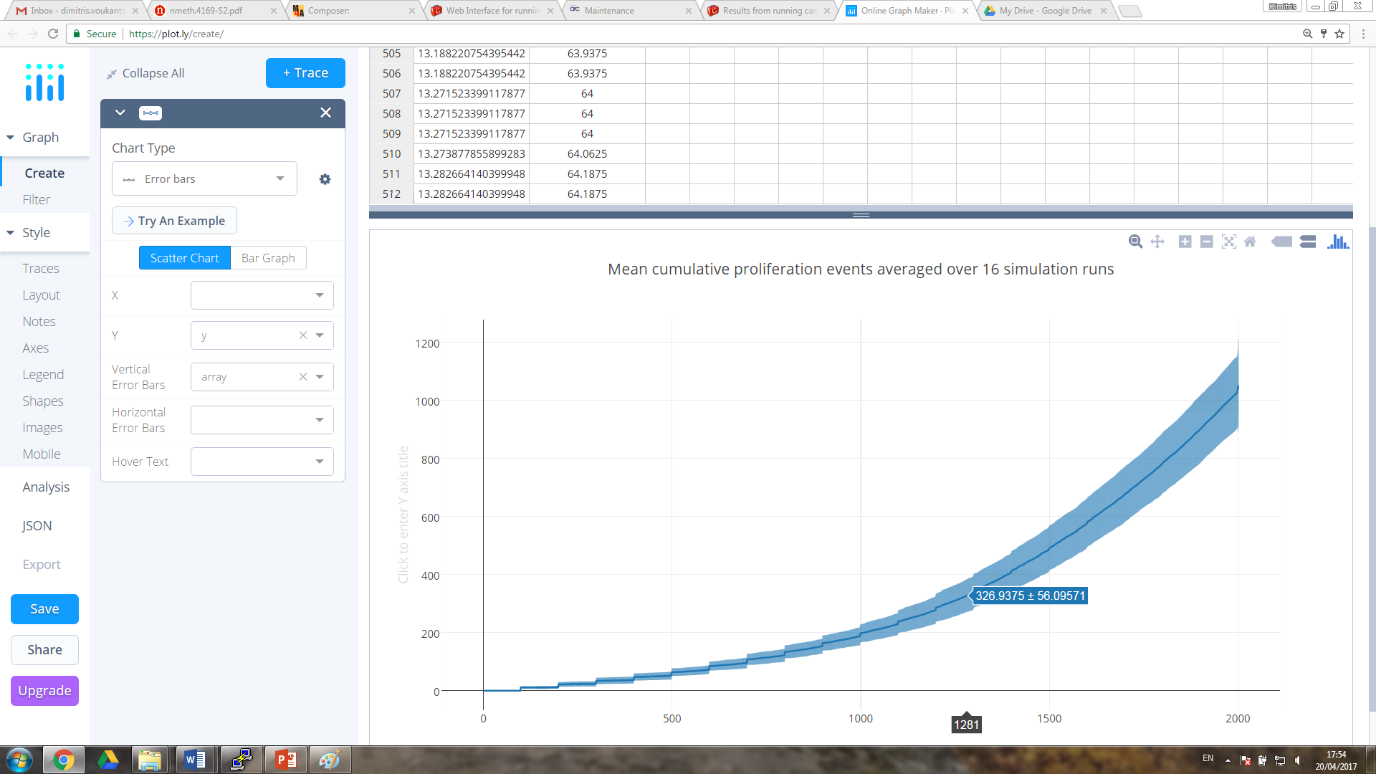
**

**Figure 7.** Plotly environment for editing figures and accessing data. The user may change the figure format and the access downlod data in spreadsheet format.

## Precompiled examples

1) Single clone experiment:

- [EGFR+, initial population: 100, replicates: 16, Time: 2000 steps.](https://merlin.oncology.ox.ac.uk/microc/run/1batches-42e4a8a1-31d2-4635-8d0f-e7bc1c824a34.html)

2) Multiple clones experiments

- [WT, EGFR+, p53-, PTEN-, p53-PTEN-, EGFR+PTEN-, EGFR+p53-, EGFR+p53-PTEN-, initial population: 100, replicates: 16, Time: 2000 steps (3D).](https://merlin.oncology.ox.ac.uk/microc/run/1batches-83b29147-2a27-4948-adf8-1f98a6129b2b.html)
- [WT, EGFR+, p53-, PTEN-, p53-PTEN-, EGFR+PTEN-, EGFR+p53-, EGFR+p53-PTEN-, initial population: 100, replicates: 16, Time: 2000 steps (2D).](https://merlin.oncology.ox.ac.uk/microc/run/1batches-cdd32f26-5f4c-4564-904f-84fa47a5400e.html)

3) Hypoxia

- [EGFR-PTEN-, initial population: 100, replicates: 16, Time: 2000 steps, Control](https://merlin.oncology.ox.ac.uk/microc/run/1batches-30205182-3dbe-41f5-a5a5-6f8bb0552524.html)
- [EGFR-PTEN-, initial population: 100, replicates: 16, Time: 2000 steps, Hypoxia](https://merlin.oncology.ox.ac.uk/microc/run/1batches-8f623e24-22e0-4e5a-a6bd-27fbb250de03.html)
- [WT, EGFR+, p53-, PTEN-, p53-PTEN-, EGFR+PTEN-, EGFR+p53-, EGFR+p53-PTEN-, initial population: 100, replicates: 16, Time: 2000 steps, Hypoxia](https://merlin.oncology.ox.ac.uk/microc/run/1batches-77a2e8d6-e853-4764-8c38-e8b413073674.html)

4) Hypoxia with signalling

- [p53-, initial population: 400, replicates: 16, Time = 2000, Normoxia](https://merlin.oncology.ox.ac.uk/microc/run/1batches-a1374753-1bea-463b-b1df-911d2bf7c44c.html)
- [p53-, initial population: 400, replicates: 16, Time = 2000, Hypoxia – no signalling](https://merlin.oncology.ox.ac.uk/microc/run/1batches-8221a999-9f59-42a9-9eb5-e908bf137eec.html)
- [p53-, initial population: 400, replicates: 16, Time = 2000, Hypoxia - signalling](https://merlin.oncology.ox.ac.uk/microc/run/1batches-bd7ac2f6-0fab-4cd7-8add-e1ba9129ef80.html)
- [WT, p53-, PTEN-, p53-PTEN-, initial population: 400, replicates: 16, Time = 2000, Hypoxia - signalling](https://merlin.oncology.ox.ac.uk/microc/run/1batches-b371fda5-d674-4233-a757-78b4d6c6cb2b.html)
