# Supplementary material for: Modeling genotypes in their microenvironment to predict single- and multi-cellular behavior
Source: Gigascience. 2019 Jan 31;8(3):giz010. doi: 10.1093/gigascience/giz010 (PMC6423375; doi:10.1093/gigascience/giz010)
Supplement: Supplemental Files [file giz010_supplemental_files.zip › Supplementary-Figures.docx]

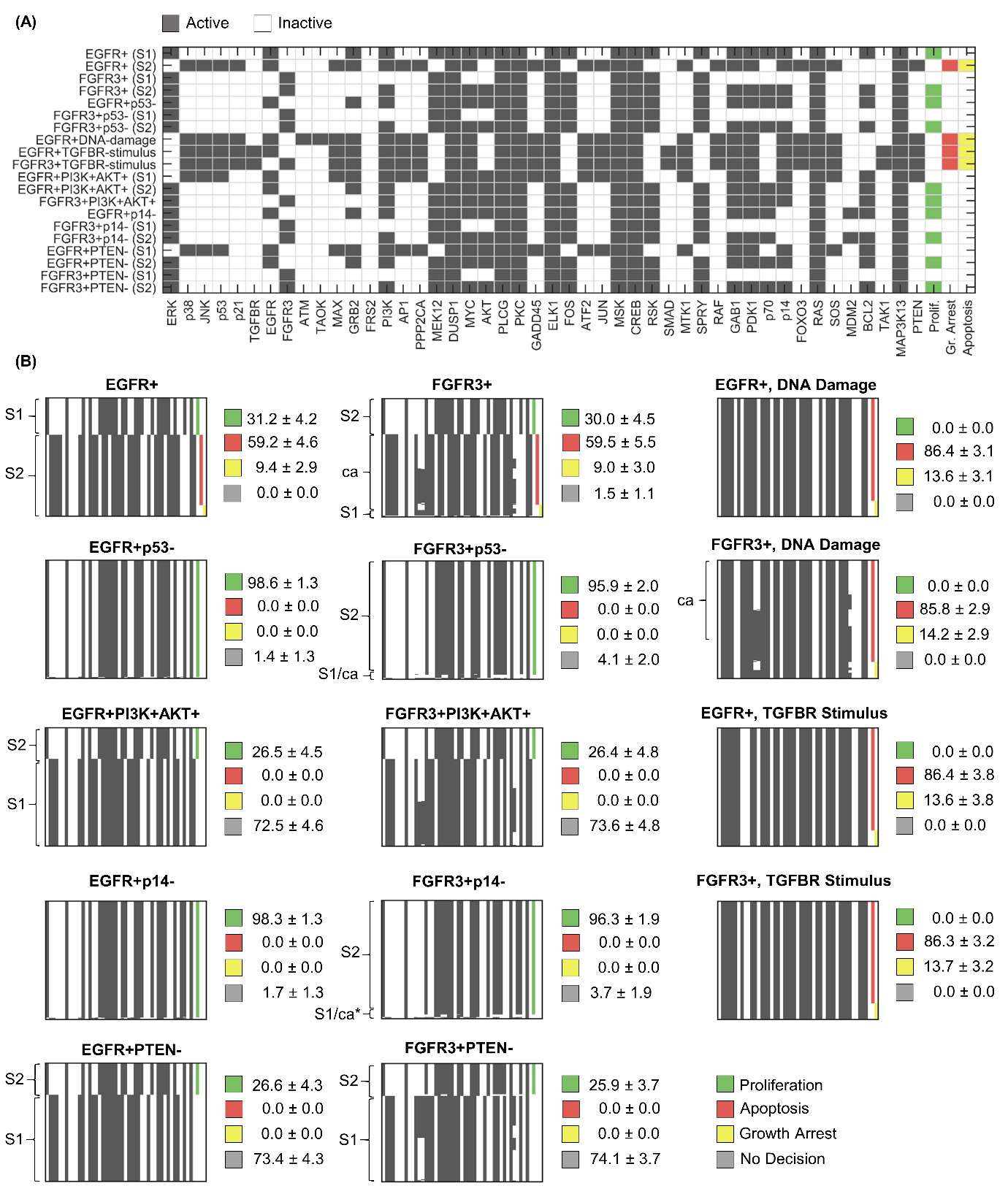


**Figure S1. microC’s ABM gene network implementation reproduces results obtained with stable-state network analysis.** We tested 14 different mutation profiles or environmental stimuli responses. (A) Stable-state analysis as presented in Grieco et al, 2013 (cyclic atractors, ca, are not shown in this graph). The horizontal axis represents the genes in the MAPK network, and the vertical axis represent stable states of different clones (S1 and S2 are multiple stable states). (B) Simulation results with microC. Each row of the heatmaps represents the state of one cell. States are composed by the activations status of genes in the MAPK network, shown in the horizontal axis in the same order they appear in Fig. A. The activation status is coded with colour: grey for activate and white for inactive genes (the last three columns are colour-coded depending on cell-fate decision). Overall there are 10,000 cells shown in each heatmap corresponding to 100 repeats with 100 cells each. The cell-fate decisions (average ± standard deviation) are shown next to the heatmaps (proliferation: green, apoptosis: red, growth arrest: yellow, and no decision: grey). (Further setup: Number of replicates: 100, Maximum number of simulation steps: 5000, Initial number of cells: 100, Cell decision window: 5001, Network update rate: 1, 3D simulation: true).


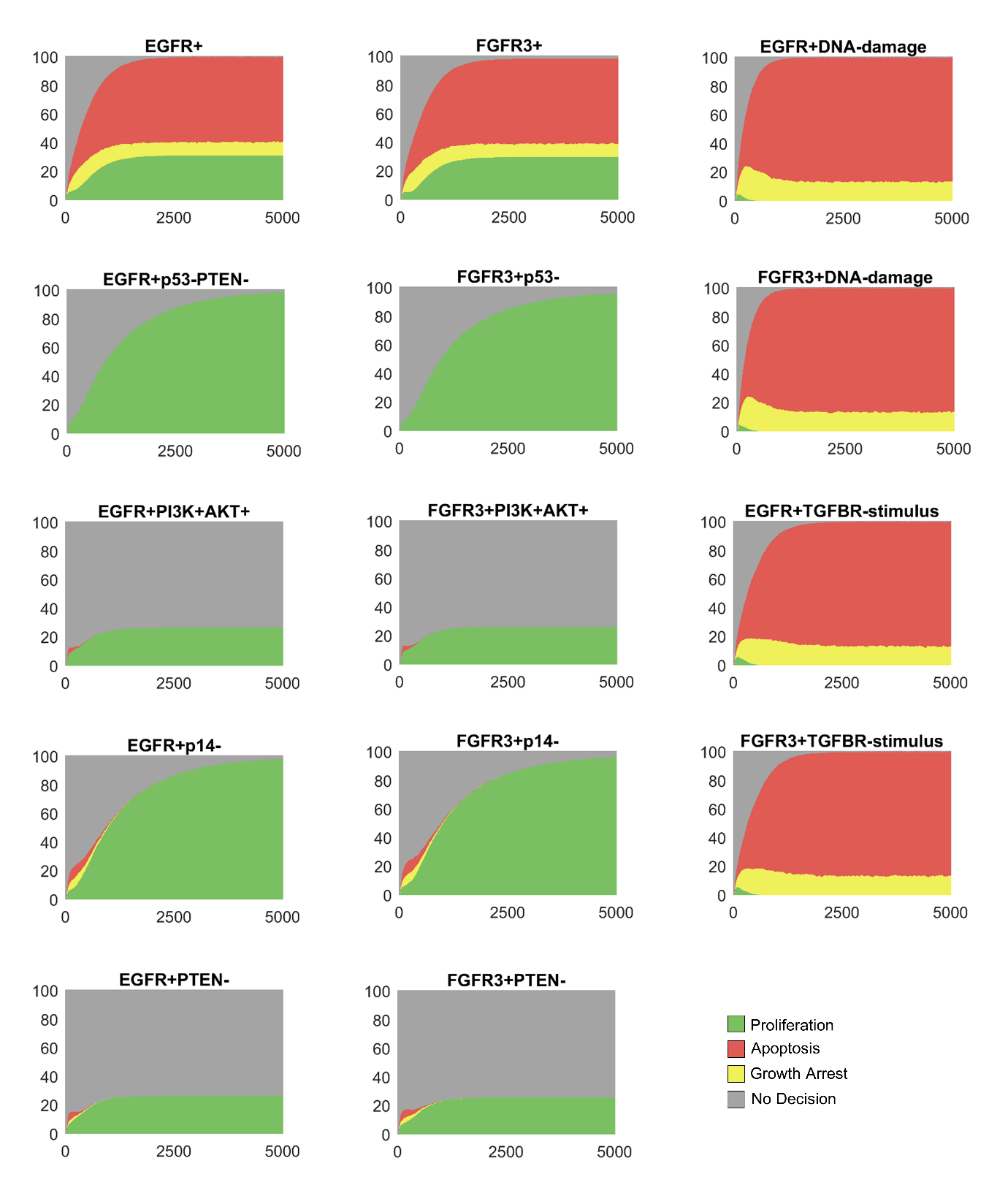


**Figure S2. Temporal evolution of cell-fate decisions for different clones.** Cell fate decisions as a function of time for the same mutations and environmental stimuli as in Grieco et al, 2013. The charts show the fraction of cell-fate decisions (green: proliferation, red: apoptosis, yellow: growth arrest, grey: no decision) in a population of 100 cells at any time point throughout the experiment (5000 temporal steps). At the end of the experiment the results have converged to the stable state analysis published in Grieco et al, 2013. Results are averages of 100 repeats. (Number of replicates: 100, Maximum number of simulation steps: 5000, Initial number of cells: 100, Cell decision window: 5001, Network update rate: 1, 3D simulation.


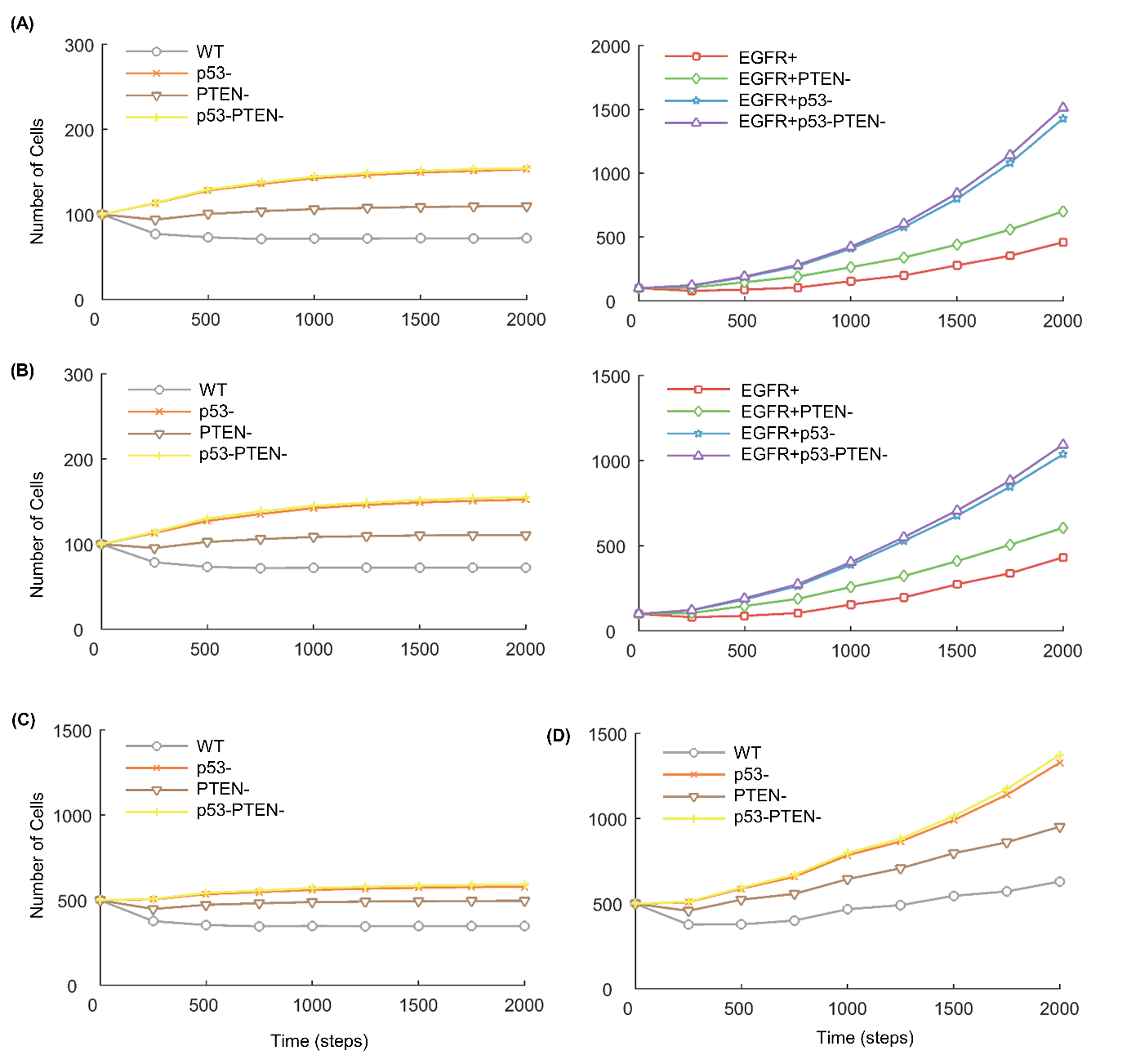


**Figure S3. Growth under Hypoxia, and Hypoxia Signalling.** Growth curves for the different clones considered in this study, under different environment conditions. (A) Growth simulating normoxia, starvation conditions (no oxygen, nor EGF diffusion), (B) Growth with oxygen level drop in the inner layer of the 3D spheroid due to diffusion (*Hypoxia*, initial and boundary condition: 0.04 mM O_2,_ R_O2_ = 5.0e-3 mM.s^-1^, d_O2_ = 1.0e-9 m^2^.s^-1^), no EGF in the media but EGF produced by the cells is diffused. (C) Hypoxia configuration with disabled EGF signalling (EGF produced but not diffused), and (D) with enabled EGF signalling. Oxygen concentration 0.04mM O_2_, ACT_EGF_ = 5.0e-4 (^+^).m^3^, R_EGF_ = 5.0e-4 (^+^).m^3^.s^-1^. Curves are averages of 100 repeats. (Number of replicates: 100, Maximum number of simulation steps: 2000, Initial number of cells: 100 (500 for hypoxia – signalling), Cell decision window: 100, Network update rate: 1, 3D simulation: true). (^+^) fraction of the EGF production rate.


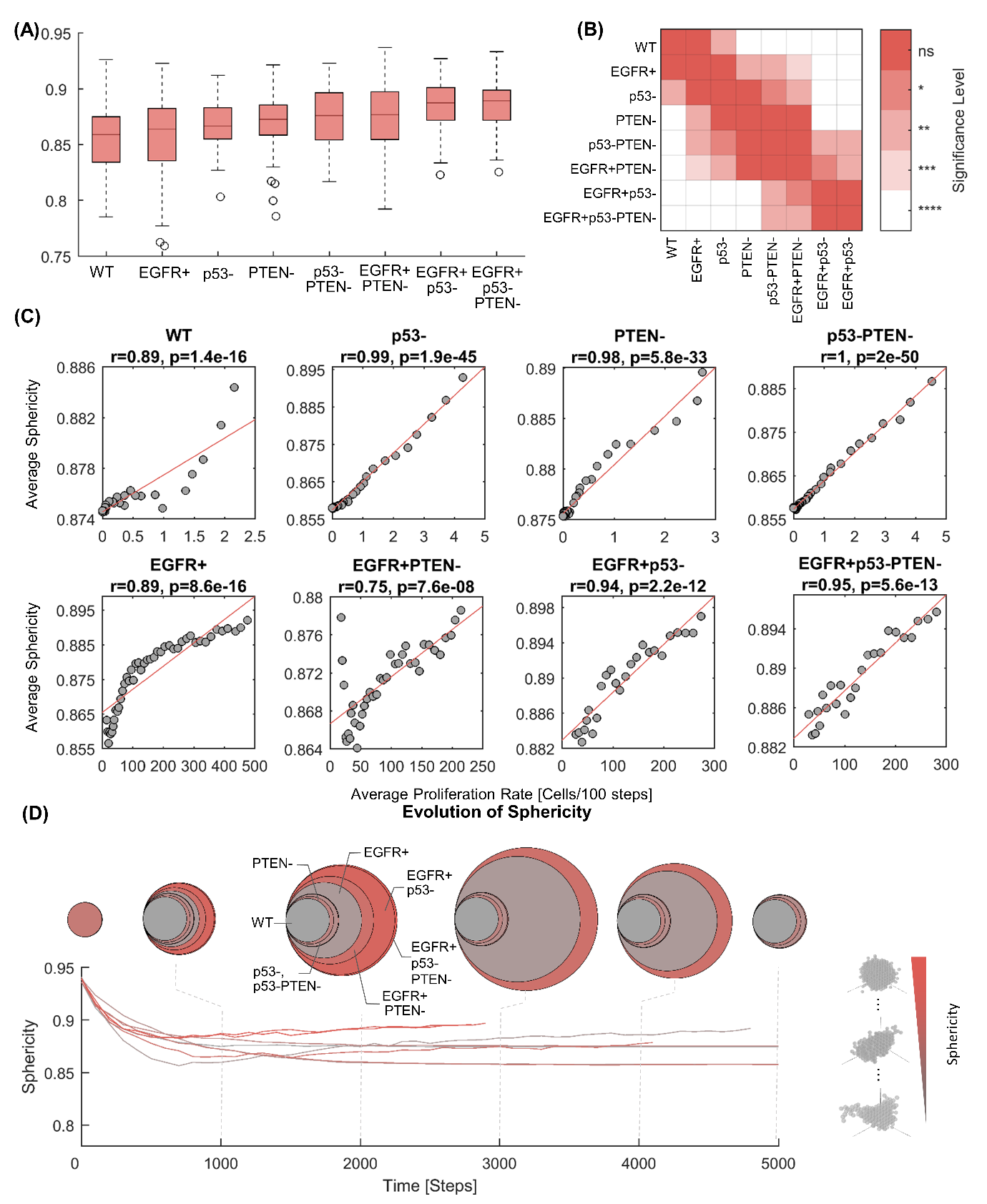


**Figure S4. Geometrical properties of spheroids depend on proliferation rate of clone.** (A) Sphericity for 100 spheroids (< 500 number of cells) for 8 different clones. (B) Significance levels for the differences observed in the boxplot. * indicates Kruskal–Wallis p<0.05, ** p<0.01, *** p<0.001, **** p<0.0001 (C) Correlation between average sphericity and average proliferation rate. We exclude the initial phase of growth (<500 temporal steps), as the cells are initially arranged in an artificial spherical setting. (D) Temporal evolution of average sphericity, and respective size of spheroids for each clone. Results are averages of 10 repeats, and spheroids limited to a maximum of 4000 cells (this is why some experiments are shorter than others).


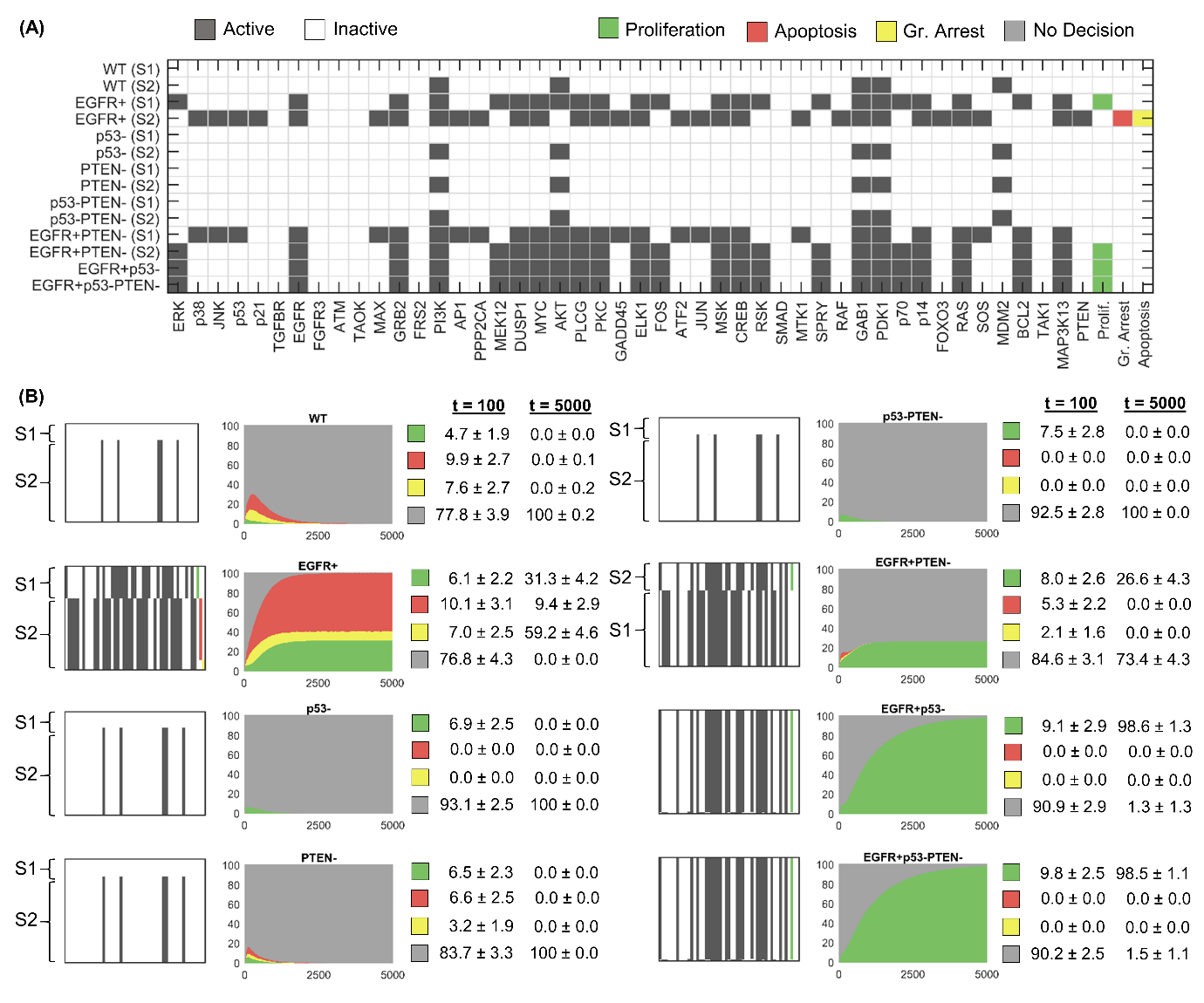


**Figure S5. Dynamical characteristics of cells with loss-of-function mutations in well-known tumour suppressor genes p53 and PTEN, and activation of the known cancer driver EGFR.** (A) Stable states analysis of clones with single or co-occurring mutations. S1, S2 refer to multiple states of the same clone. Colours in the last three columns correspond to cell-fate decisions (proliferation: green, apoptosis: red, growth arrest: yellow). (B) Heatmaps show the activation status of genes in MAPK network (horizontal axis). Each row of the heatmap represents the state of one cell. Overall there are 10,000 cells (100 repeats with 100 cells each). The stacked bar charts show the fraction of cell-fate decisions at the cell population (100 cells) at any time point during the experiment. The results are averages of 100 repeats. The cell-fate decisions (average ± standard deviation) are shown next to the heatmaps for two distinct time points: 100 steps after the experiment starts and, at the end of the simulation. (proliferation: green, apoptosis: red, growth arrest: yellow, and no decision: grey). Number of replicates: 100, Maximum number of simulation steps: 5000, Initial number of cells: 100, Cell decision window: 5001, Network update rate: 1, 3D simulation: true.


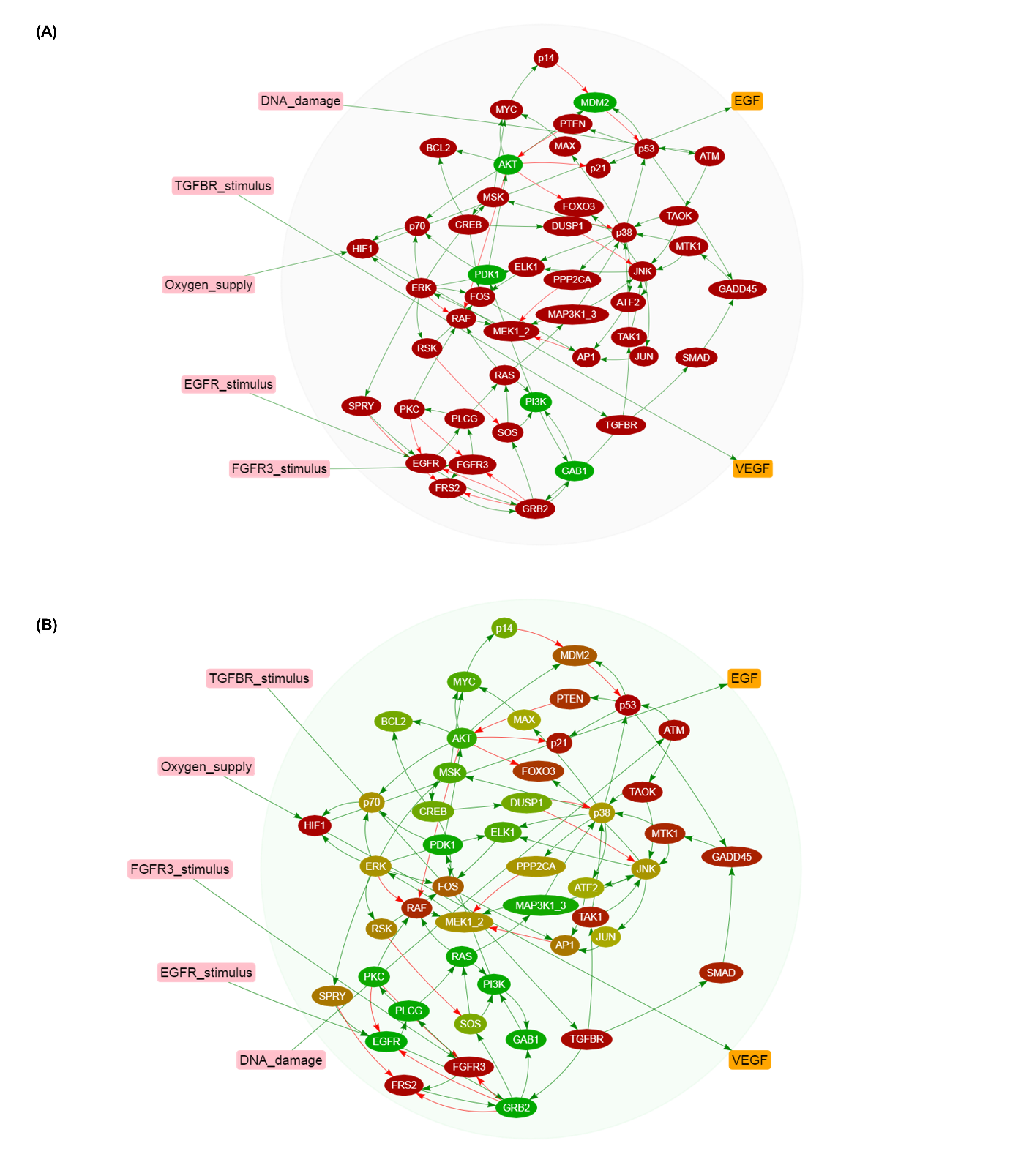


**Figure S6. Activation status of genes is determined by mutation profile**. The MAPK network used in the simulations in both of these cases is the same, yet the mutations profile different. (A) WT cells, and (B) EGFR+p53-. The circle represent the cell boundary. Inside the circle: network nodes represent gene products (colour represents activation status, red: inactive, green: active); Edges represent interactions between genes (red: inhibition, green: activation). Outside the circle: Pink nodes (left) represent receptors (linked with environmental stimuli), and orange nodes (right), represent output nodes (cell products).


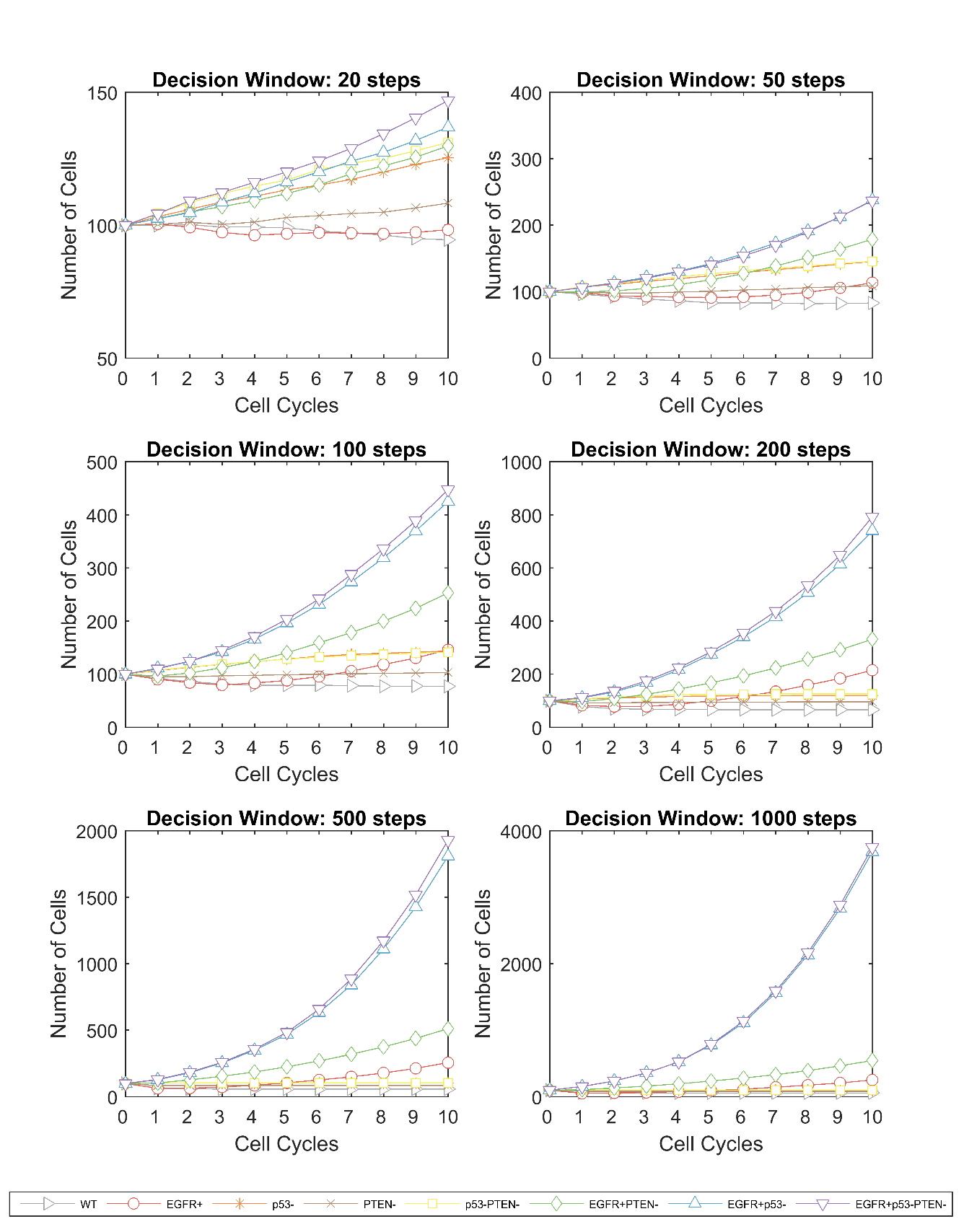


**Figure S7. Sensitivity analysis for the effect of the multiscale parameter *cell decision window* on growth curves.** Growth curves of clones under the same experimental condition, but with different values for the parameter cell decision window (the simulation “cell cycle” time), which regulates the timing of extra-cellular actions, such as cell-fate decision, with respect to the timing of events internal to the cell, determined by the network execution (shown in the horizontal axis). Curves are averages of 10 repeats that run for 10 cycles. A decision window as small as 100 was sufficient to replicate the same qualitative results (exponential growth for EGFR over-expressed clones, smaller growth for non-EGFR over-expressed clones) that we observed with much higher values of the cell decision window, thus decreasing the simulation time by a factor of 10.


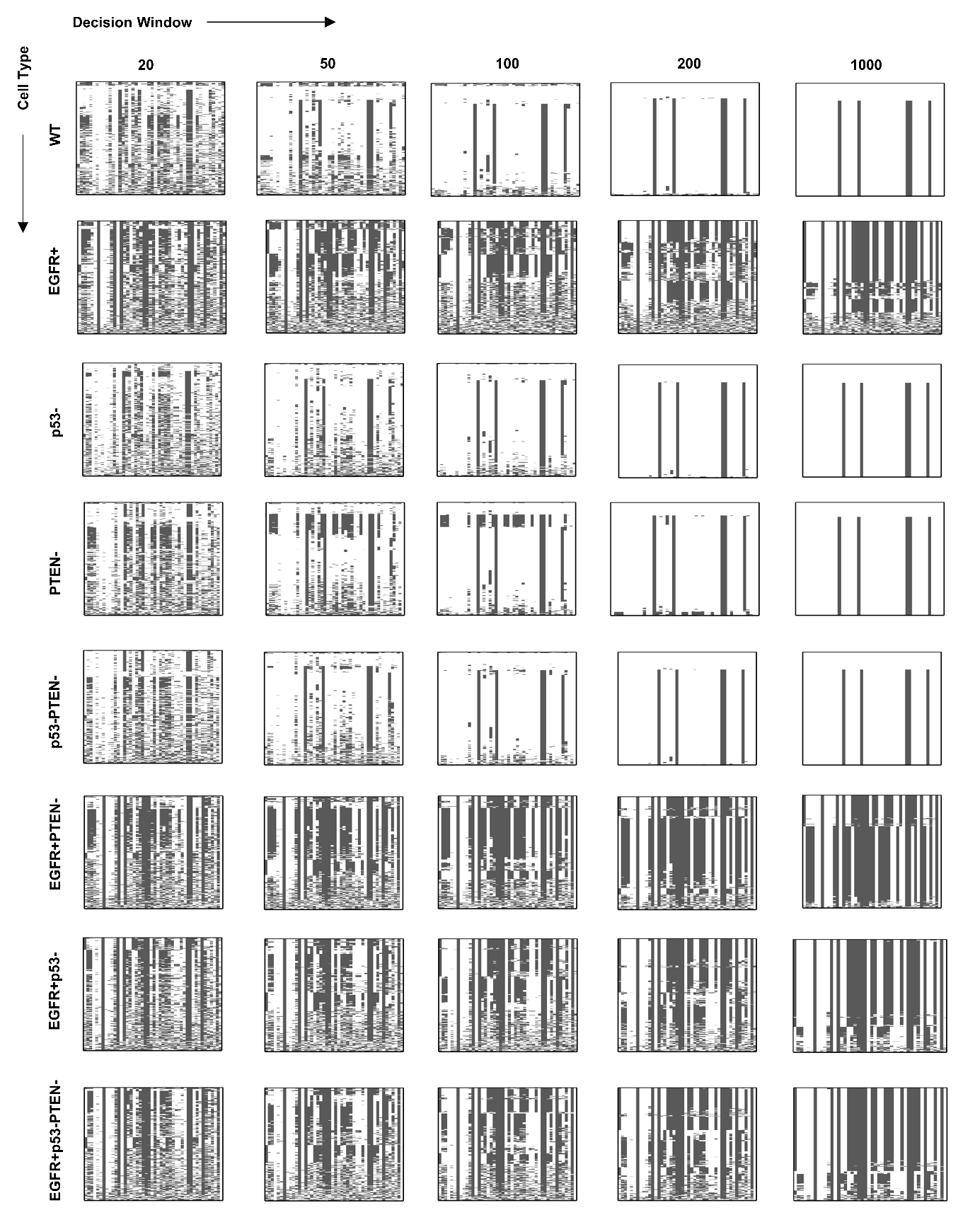


**Figure S8. The sensitivity of microC state activation/inactivation when changing the value of the decision window paramater.** The decision window is the number of internal (network) steps for every external (inter-cellular) step. Longer internal simulations (large decision window) result to network stable states (figures on the right). Each row of the heatmaps represents the state of one cell. States consist of the activations status of genes in the MAPK network, shown in the horizontal axis. The activation status is coded with colour: grey for activate and white for inactive genes (the last three columns are colour-coded depending on cell-fate decision). Overall there are 10,000 cells shown in each heatmap corresponding to 100 repeats with 100 cells each.


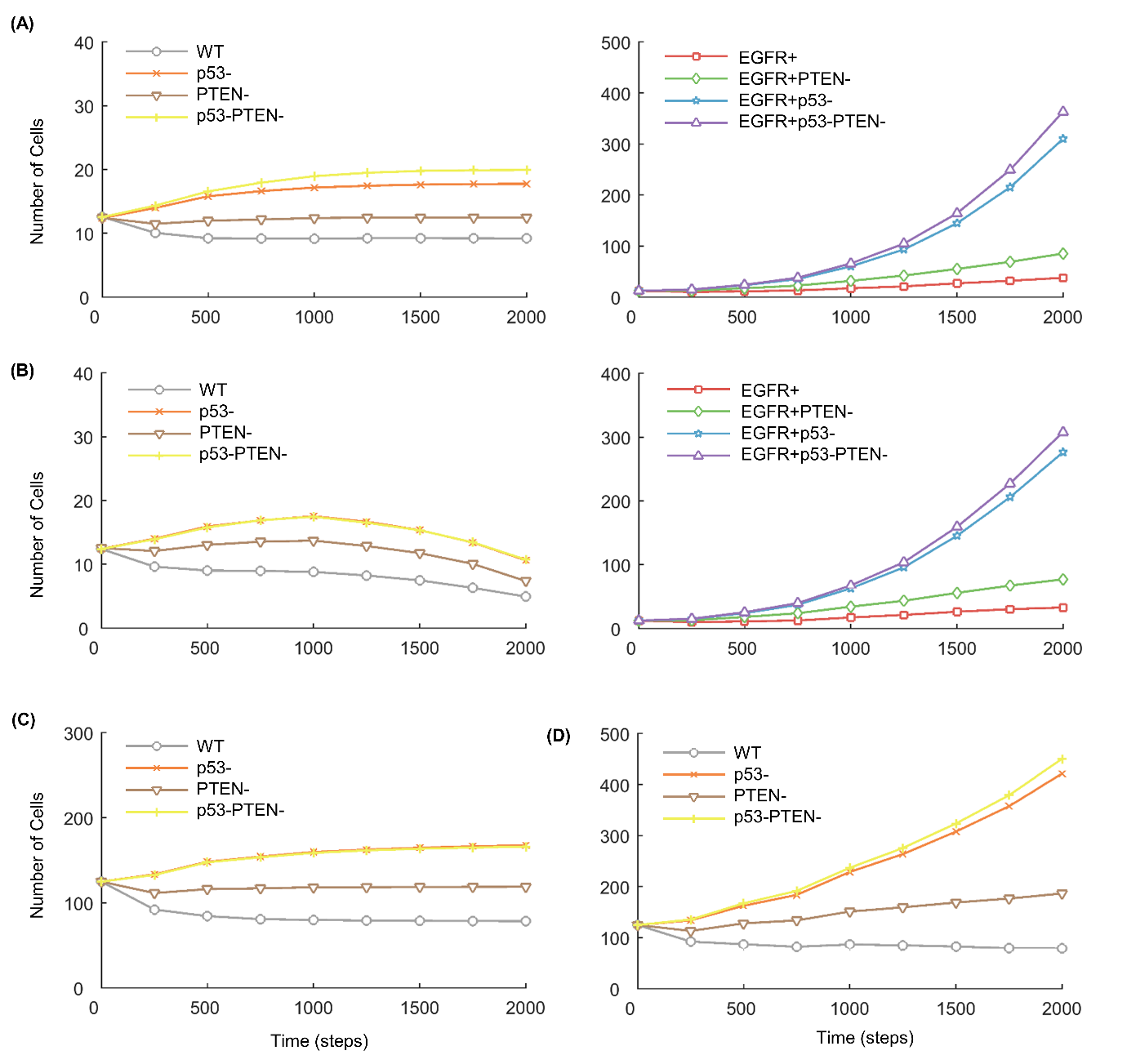


**Figure S9. Growth under competition in Hypoxia, and Hypoxia Signalling.** We studied growth for 8 different mutation profiles grown under different conditions; in Figure S3 we considered these as monoclonal spheroids, here we grow the clones together in multi-clone spheroids and we study competition between the clones. (A) Growth under competition without considering oxygen diffusion, (B) Growth under competition with oxygen level drop in the inner layer of the 3D spheroid due to diffusion (*Hypoxia*, initial and boundary condition: 0.04 mM O_2_), (C) Growth under competition and hypoxia configuration with disabled EGF signalling, and (D) with enabled EGF signalling (Oxygen concentration 0.04mM O_2_, ACT_EGF_ = 5.0e-4 (^+^).m^3^, R_EGF_ = 5.0e-4 (^+^).m^3^.s^-1^). Curves are averages of 100 repeats. Interface setup: Number of replicates: 100, Maximum number of simulation steps: 2000 (5000 for hypoxia – signalling), Initial number of cells: 100 (500 for hypoxia – signalling), Cell decision window: 100, Network update rate: 1). (^+^) fraction of the EGF production rate.


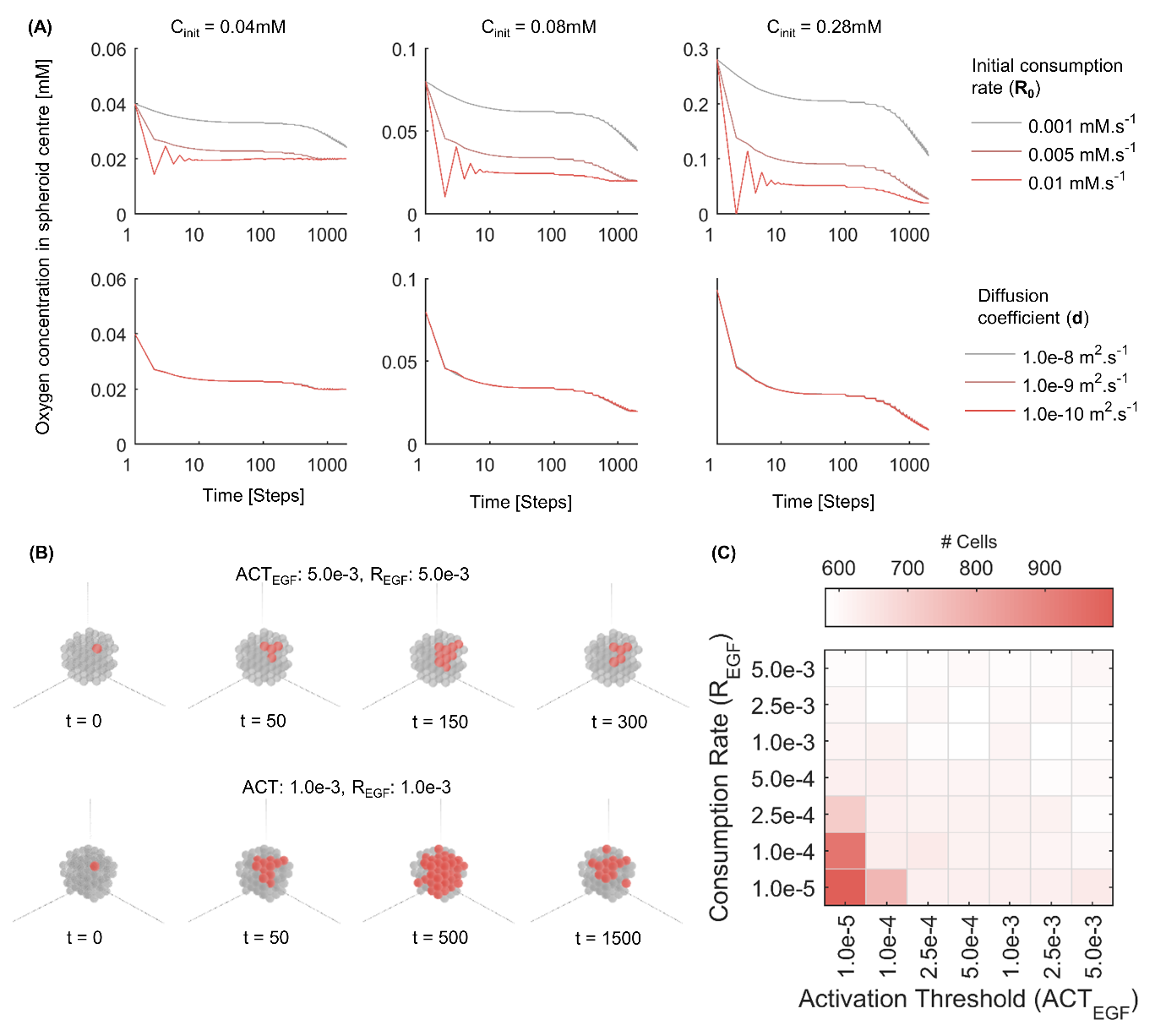


**Figure S10. Sensitivity Analysis for the parameters regulating the diffusion processes.** (A) Extreme low oxygen concentrations (0.02mM O2), may trigger a necrotic response to cells. Here, we demonstrate the oxygen concentration at the centre of growing spheroids for various values of the initial Oxygen consumption rate (R_0_), and the Oxygen diffusion coefficient (d). The experiments are repeated for three different values of the initial oxygen concentration (C_init_). The line are averages of 10 repeats. (Initial population: 100 cells, 2000 temporal steps). (B) Intensity and duration of an EGF response starting at a single spatial point. The effect is regulated by the activation threshold for EGF receptors (ACT_EGF_) and the consumption rate of EGF (R_EGF_). Red-coloured cells receive a sustained EGF stimulus from their environment. (C) Growth under different combinations of the Consumption Rate and the Activation Threshold. Colour represents the number of cells (results are averages of 10 repeats, initial population: 500 cells, clone: p53-PTEN-).
