# Supplementary material for: Modeling genotypes in their microenvironment to predict single- and multi-cellular behavior
Source: Gigascience. 2019 Jan 31;8(3):giz010. doi: 10.1093/gigascience/giz010 (PMC6423375; doi:10.1093/gigascience/giz010)
Supplement: Response_to_Reviewer_Comments_Original_Submission.pdf [file giz010_response_to_reviewer_comments_original_submission.pdf]

Author's Response To Reviewer Comments

Close

Dear Dr Nicole Nogoy,

We thank you and the reviewers for considering our manuscript.

The comments provided have enabled us to significantly revise our manuscript, and we are glad that in general, the reviewers agree with the quality of our work. We do also appreciate that they have raised some important concerns, and we address each of these in the point-by-point response below. We feel that the review process has brought up very useful critics, and extremely constructive suggestions which allowed us to significantly improve the manuscript.

In the revised manuscript, we have specified all key aspects of the modelling framework, and have added comparison with experimental data reflecting our modelling choices. This enabled us to discuss clearly both strengths and limitations of the framework. In addition, we have followed the editorial suggestion of registering our software application in the SciCrunch.org database and received a RRID (Research Resource Identification Initiative ID) number, which is now included in the manuscript on the abstract page for future tracking and reproducibility.

The comments of the reviewers (delimited by •• ••), and a point-by-point reply are shown below each comment. We have tracked all additions and changes in the manuscript file. We feel very strongly that the resulting revisions have increased the readability of the paper, and the usefulness and accessibility of our framework.

Thank you for your consideration of our significantly revised manuscript and formal rebuttal.

Yours truly,

Francesca Buffa & colleagues

Reviewer #1:

•• It is not clear at all how authors prepared samples (dataset or algorithm) to analyze. They wrote "Specifically, we consider a population of homogenous pre-cancerous cells growing in a 3D environment; then, we gradually introduce mutations in onco- and tumour suppressor genes (Figure 1A) to study how they affect the multi-cellular growth (Figure 1B)." And Materials&Methods show "Cells as computational agents: Cells are represented in microC as computational agents with a physical dimension. Cells may interact with the microenvironment by consuming oxygen, and with other cells, via diffusible substances, such as cytokines, chemokines, growth factors, and hormones, whose production is defined by the network dynamics. This can be customised by the user. Furthermore, cells are also meta-agents, namely each cell is itself populated by a community of computational agents, the genes and molecules,

which act and interact as a network inside the cell agent." These do not indicate how authors prepared each parameter. Were original biological models from cell lines or cells from genetically modified mouse mammary tumor or primary cells from patients? ••

This is a very important point and in reading the manuscript again, we do agree that this description was not sufficiently clear. We thank the reviewer for this critical comment. We have now extensively changed the manuscript to clarify what are the inputs and parameters that the framework use to fully specify a model, and also the choices made in the specific models developed and simulated in the manuscript. We indicate all changes (and relevant parts of the manuscript which were modified) below.

The model is defined by the choice of the network (input file: regulatoryGraph.html), the mutation profile (input file: mutations.txt), cell-environment and cell-cell interactions (input files: assotiations.txt, input-parameters.txt, diffusion-parameters.txt). In addition to the parameters defined in these files above, there are 10 parameters required by the user via the web interface of microc. These are:
1. Number of replicates
2. Max number of steps
3. Maximum number of cells
4. Initial number of cells
5. Cell decision window
6. Network update rate
7. Cell diameter (relevant only when diffusion processes are taking place)
8. Diffusion update rate (relevant only when diffusion processes are taking place)
9. Grid sparsity (relevant only when diffusion processes are taking place)
10. Simulating environment (2D or 3D)

We have now extensively revised the manuscript, and specifically the sections suggested by the reviewer (Methods: Cells as computational agents, Methods: Cell mutation profiles), to include further information on this.

We have also spelled out the details of the modelling in the Introduction to clarify the scope, and we have added an overall description of the parameters in the very first paragraph of the method section Methods: Key elements of the framework. This helps navigating the rest of the results, methods and supplementary protocol, where the results are presented and the structure of files and the function of each parameters are described. Further description can be also found in the documentation of microc.org. We have now spelled this out.

In the specific example studied here, we have chosen breast cancer as a model. Thus, the mutations reflect those frequently found in these cell lines. We have now justified this choice, and introduced these details in the manuscript in the Introduction and in the section Results: In-silico growth of a heterogeneous population of cells communicating within a three-dimensional microenvironment (first paragraph). The choice for the Oxygen and EGF signalling parameters were based on previous work (Jiang et al, 2005) and it is not specific to breast cancer cell lines. However, we have carried out a sensitivity analysis to understand the implications of these choices on our model predictions. This is shown in Figure S10.

Finally, we acknowledge that the values used for each experiment were not clearly stated in a single point, so they were not always easy to follow. To address this, we added a Table (Table S1) where we reported all the values used for each parameter, in each model and simulation experiment.

•• And what is this "physical dimension" and "physical (micro) environment? Physical (micro)environment usually indicates mechanical force from microenvironment, which is not well described and discussed in this manuscript. I guess that authors want to refer "tumor microenvironment" and its each content (cell-ECM, cell-cell, chemical and physical property) as their "dimensions". If so, this "physical dimension" and "physical (micro)environment" are not proper definition in this study. ••

Indeed, the term “physical” was used by us in a too general manner, and we agree that this could be misleading and cause confusion. This was correctly noticed and pointed out by both reviewers. We have considered the suggestions given by both reviewer on how to address this point, and we have replaced the term “physical microenvironement” with either just “microenvironment” or “tumor microenvironment” as required by the specific context. These changes are tracked throughout the manuscript.

We have also clarified this aspect in the section Methods: Cells as computational agents, where we now spell out precisely which are the specific aspects of the physical microenviroment that we take into account. Namely, the three-dimensionality of the model, the presence of neighbouring cells, and local chemical concentrations.

We thank the reviewer for this comment. As a result, our statements are now much more specific and describe the specific physical aspects considered.

•• WT sample indicated on Figure2C shows mass of cells, and the other indicated on Figure 4B shows scattered single cells without cell-cell association (epithelial polarization). Why these showed totally different predictions for 3D morphology? Can this predict normal tissue development? If so, this WT (with no mutation) should show normal epithelial morphology which is known as "acini" or ductal tubules. In current manuscript, there is no "normal" tissue compared. Therefore, it is not clear if this prediction is working fine or not. If this "WT" indicates a tumor with no mutation, then what kind of microenvironment induce these scattered (with less growth) or mass of cells? ••

Thank you for this question. We agree that this was not clear in the manuscript. In the model shown here, WT are pre-malignant cells. The structure shown in Figure 4B is the 3D growth of these pre-malignant cells when grown in a mixed population spheroid. Specifically, the figure was meant to show a spatial decomposition of a multiclonal spheroid. This was simply to illustrate the distribution and abundance of each population of clones in the spheroid, similar to the 2D example shown in Figure 4A. We modified the plots in Figure 4B to illustrate this

clearly, and we corrected the corresponding legend to clarify this.

•• P53 mutations are variable. Can this system analyze the effect of mutant p53 functions and compare WT and p53-null conditions? And then what is the difference between these?••

In these simulations, we are investigating p53-null, or loss-of-function, with respect to p53 WT conditions. As the reviewer is correctly pointing out, there are a large number of p53 mutations which do not cause loss of function but instead a change of function. The functional consequences of some of these mutations are well understood, others less so.

We have now extensively clarified the specific mutations included in this first model (Results, first section), discussed experimental models carrying the specific mutations (Results, second section), the limitation of the current choices and the capabilities of our modelling framework (Discussion). Specifically, the agent based modelling core of microC allows to consider different types of mutations in the form of different ABM rules, which can be coded in the network simulated by microC. While it would be outside the scope of this manuscript to conduct a systematic assessment of the functional implications of p53 mutations (several not yet well understood), this comment has pointed out a very important aspect and we have now clarified both the specific scope of the current study and the methodological potential of the framework (Discussion).

•• Cancer progression include invasive conversion. Can this program predict invasive phenotype in 3D system? In current manuscript, it seems that only cell growth and cell death/necrosis are highlighted. Therefore, the invasiveness might have been ignored in this work. ••

The current model considers proliferation and not migration as a potential outcome. We have now modified the manuscript to specify this current limitation and its implications, and motivate our choice. Specifically, we discussed the modelling choice not to include migration as cell decision in Methods: Cell status and cell-fate decision rules, and in the same section and the Results we discuss the implications, and how this applies to the particular problem simulated. On the other hand, we acknowledge that the migratory potential of certain cell types is an important parameter to study for future development (Discussion).

•• "Hormone" is used as a parameter in this study. However, signaling from hormone is depending on the levels of hormone and hormone receptor. Are these parameters correctly used in this system? So far, hormone diffusion is only taken in account, meaning that the authors only consider the condition in the presence of hormone. Can the prediction also compare with or without hormone and hormone receptor? ••

Yes, the reviewer is correct. We did refer to hormones as one of the examples for diffusible substances which may be produced and consumed by cells. In this specific study, we demonstrated this effect using the example of EGF signalling (section 6). Essential parameters

for this function are: the initial concentration of the substance, which may be set to simulate presence/absence of substance, and the consumption/production rates of the diffusible substance, which in microC are step functions defined by the user. The choice of these rates determines the strength and the duration of the cell-cell interaction (we show a sensitivity analysis for this choice in Figure S10). In order to clarify this aspect, we have extended the description of the methods used for modelling cell-cell interaction via diffusible substances in the section Methods: spatially-aware environment.

With respect to the second part of the question, we have simulated cases where EGF is present or not present, and compared results, in Figure 5E-G. Namely, in the presence of hypoxia EGF is produced by the cell and diffused to neighbouring cells. In the normoxic conditions simulated there, no EGF is produced and thus EGF is not present in the environment (no other background EGF introduced the media for this simulation).

Importantly, in our model we can interfere with the signalling activation. In the case of EGF, EGFR can be activated upon EGF binding, or be constitutively activated by mutations (EGFR+), or be inactive by loss-of-function mutation. However, we can also regulate the presence or absence of the actual growth factors, simulating for example conditions of starvation (e.g. EGF can be present and diffused, mutated and diffused, or depleted). This means that we can both simulate a drug affecting the receptor function, or a knock down experiment where the growth factor is knocked out. All this was not clearly stated in the manuscript, and it is indeed an important point. This is now clearly explained in the text (Results: Interaction between the genotypes and the physical tumor microenvironment affects multi-cellular growth and clonal selection, third paragraph). We thank the reviewer which has enabled out to point out this important aspect.

•• This may be the most important point to understand the significance of this study. If the actual biological 3D culture assay with these cancer cells with different levels of EGFR, PTEN and P53 is performed, does the predicted 3D morphology (and its growth pattern of each clone) in this simulation match to an actual 3D cell culture result? And the difference between actual 3D growth of cancer cells and predicted 3D morphology should be discussed well in this manuscript. At least, 'real' 3D culture result should be tested and compared. ••

This manuscript aims at introducing the microC modelling framework, hence the specific model was intended to be explicative of the modelling capabilities. However, we do agree that it is important to establish the usefulness of this first model, and we have now compared our predictions from this first implementation, and the specific example, with experimental data describing alteration of the specific mutations simulated.

We have introduced a comparison of the growth curves from our simulations of the different clones with experimental growth using cell lines carrying the specific mutations we have modelled, and grown under similar starvation conditions. This comparison is reported in the Results: Effect of mutations on gene network dynamics and multi-cellular growth, third paragraph.

Additionally, we present a comparison with experiments looking at doubling time in the context of EGFR activating mutations, again providing support for the model predictions. This is now presented in the Results: Effect of mutations on gene network dynamics and multi-cellular growth, fourth and fifth paragraph.

Furthermore, throughout the results and discussion we have highlighted all cases in which we have either discrepancy with experimental results, or no direct experimental validation at the moment, as the necessary experiments would be outside the scope of this study. Finally, to address this and the above points made by this reviewer, we have improved the overall presentation of the results throughout the manuscript, with a greater amount of detail on the specific parameters and models chosen, and more relevant figure display (see for example modified Figure 1).

We thank the reviewer for this comment and feel that the modified results presentation and discussion is fairer and it allows to better appreciate the goodness and limitations of the current model, while highlighting areas and specific ideas for further research.

Reviewer #2:

•• In the manuscript "Modeling genotypes in their physical microenvironment to predict single- and multi-cellular behavior," Voukantsis and colleagues present an open-source, agent based modeling framework for modeling the behavior of cells in three dimensional environments. Agent decisions (at the cellular level) are based on activation status of prescribed gene networks, which drive cellular behaviors, namely proliferation, necrosis, apoptosis, or quiescence. These behaviors lead to emergent and time-dependent properties that are monitored.

The ability to model and interrogate cell behavior at the single-cell level and incorporate mutations and heterogeneity in the model will make it a useful tool for understanding cell proliferation in three dimensions. As the authors note, using the model in combination with single-cell imaging and sequencing methods could provide important insights into how underlying genetic/cell signaling activity influence cell phenotype. The reviewer envisions that the model, in its current implementation, will be most useful to experimentalists studying cell spheroid growth in various three dimensional cell culture matrices and for drug discovery applications aimed at inhibiting primary tumor growth or stem cell survival/expansion.

Due to some limitations in the modeling of the physical microenvironment in the current implementation of the model, the reviewer suggests minor revisions, primarily in language used to describe the model, prior to publication. ••

Thank you for the positive review of our work, and the very useful comments. We have addressed each comment in the points below.

•• Major comments:
1) The authors emphasize that the model can "account for the effect of the physical microenvironment." While certain aspects of the physical environment are accounted for, including the location of the cells in three dimensions, the presence of neighboring cells, and local chemical concentraitons, other aspects are not included. Notably, physical factors such as matrix porosity, matrix stiffness, and topographical cues are not included. These physical cues are important in cell behavior (see, for example, Haeger et al., Biochimica et Biophysica Acta, 1840(8):2386-2395 (2014)). Attempts have recently been made to model some aspects of these physical cues (Ahmadzadeh et al., PNAS 114(9):E1617-1626 (2017)). While the absence of these physical cues in the manuscript does not invalidate the model, the physical aspects that are included should be clarified, and caveats about what is not modeled should be made. Instead of stating that the model accounts for the physical
microenvironment, the authors should state that the model accounts for certain important aspects of the physical microenvironment. This could look similar to the second statement in the "Cells as computational agents" section. ••

Indeed, the term “physical” was used by us in a too general manner, and this could be misleading, as pointed out from the first reviewer as well. We have considered both comments and replaced the term physical microenvironment with either just “microenvironment” or “tumor microenvironment”, as required. This makes our statements more specific. We have also clarified this in the section Methods: Cells as computational agents, where we now spell out which are the specific aspects of the physical microenviroment that we take into account. Namely, these are the three-dimensionality of the model, the presence of neighbouring cells, and local chemical concentrations.

•• 2) On a similar note, one particular cell behavior that is particularly important to cancer biology, namely cell motility, is not included as a potential cell behavior in the model. Particularly in the case of cell spheroid growth, the "go or grow" dichotomy that dictates whether cells in a spheroid divide or migrate out of the spheroid is of interest. See, for example, Garay et al., Experimental Cell Research 319(20):3094-3103 (2013) and Jimenez et al., Oncotarget 6(41):43438-43451 (2015). The authors should make it clear that cell migration was not considered in the model and indicate situations where this assumption is most appropriate. ••

Thank you for the comment, which was also made by the first reviewer. We have now specified both the current limitations of the specific model presented, and methodological considerations regarding the modelling framework. Specifically, we highlighted the modelling choice not to include migration as potential cell decision at this stage (Methods: Cell status and cell-fate decision rules, last line), and we then discussed how this applies to the particular problem simulated and the future potential of the methodology (Discussion, but last paragraph).

•• 3) Page 6, line 1 - "Cell-fate decisions are considered to be terminal, namely once they are triggered the network simulation fo the gene network stops." Does this mean that cells that enter

a profliferative state, for example, proliferate indefinitely? Or are there multiple simulations of the gene network at each time step before a cell fate decision is made? Please clarify here. It appears that this issue is addressed in part in section 3 of the results, but it would be helpful to emphasize it here.••

Thank you for pointing this out. Cell fate decisions can in fact change in between cell actions. What cannot change is the action once it is made.

Cells that have already proliferated re-enter the simulation for another round after resetting the cell-fate node, so that any outcome is possible in the new round. Although cells can in principle proliferate multiple times if the cell-fate decision node is activated again, there are limitations to how fast this can happen (determined by the parameter “decision window”) and how many times due to availability of neighbouring free slots, i.e. if there is no empty slot the proliferation action won’t happen and the cell will enter into growth arrest.

We have updated the text in Material and methods: Cell status and cell-fate decision rules to describe more accurately these aspects of the modelling.

•• Minor comments:
1) The supplementary user guide and supplementary figures are nicely done. In particular, the sensitivity analyses in the supplementary figures are very informative and are presented well.
2) Page 8, line 19 - typo in "framework"
3) Page 9, line 9 - should state "three possible decisions"
4) Page 13, lines 1-9 is a nice example of how changing environmental cues can effect cell phenotype and tumor heterogeneity.
5) Page 13, line 27 - "section 4" should read "section 5."••

Thank you. We have updated the text accordingly for each of these.

•• 6) Page 14, line 36 - which other networks besides the MAPK network does microC come with? What applications would these other networks be useful for?••

At the moment the MAPK network presented is the default network used in microC. We discuss some possible extensions, but the users may introduce their own network. This is was only mentioned in the methods, but it is now spelled out more extensively and we have added a comment also in the Discussion.

Close
