# Supplementary material for: Modeling genotypes in their microenvironment to predict single- and multi-cellular behavior
Source: Gigascience. 2019 Jan 31;8(3):giz010. doi: 10.1093/gigascience/giz010 (PMC6423375; doi:10.1093/gigascience/giz010)
Supplement: Response_to_Reviewer_Comments_Revision_1.pdf [file giz010_response_to_reviewer_comments_revision_1.pdf]

Author's Response To Reviewer Comments

Close

Dear Dr Nogoy,

thank you for considering our manuscript. We have reviewed the manuscript, and provided all the information requested. Point by point response is included below. We worked on the edited version of the manuscript you have sent to us, and we are resubmitting the modified version. We hope that this reviewed manuscript can be accepted for publication.

Yours sincerely,

Francesca Buffa

Point by point reply (replies in capitals):

1. Please add ORCID IDs for all authors on the title page - if you do not have one, please register at https://orcid.org/
2. For reproducibility of methods, we ask our authors to add protocols in Protocols.IO. Please add the MicroC protocol in Protocols.io and cite the Protocols.io DOI in the manuscript. Note that you can add a more simple version giving an overview of the steps and include the MicroC Protocols PDF with it as a more detailed version.

WE HAVE ADDED THE INFORMATION ON ORCID AND PROTOCOL IN THE MANUSCRIPT, AND MODIFIED THE PROTOCOL AS REQUESTED, THANK YOU

3. As agreed by email prior to peer review, please make all the source code, documentation plus any scripts available in GitHub and add the following missing sections in the paper:

WE HAVE CHOSEN A OSI LICENSE, PROVIDED THE INFORMATION AND SOURCE CODE LINK ON THE MAIN MICROC PAGE, STATED CLEARLY BOTH THE OSI LICENSE AND THAT THE SOFTWARE CAN BE USED WITHOUT A LOGIN, AND MODIFIED THE MANUSCRIPT WITH THE ADDITIONS AS SUGGESTED BELOW

4. Add the following sections:

A) Availability of Source code and requirements:

Project name: e.g. My bioinformatics project
Project home page: e.g. http://sourceforge.net/projects/mged
Operating system(s): e.g. Platform independent
Programming language: e.g. Java
Other requirements: e.g. Java 1.3.1 or higher, Tomcat 4.0 or higher

License: e.g. GNU GPL, FreeBSD etc.
Any restrictions to use by non-academics: e.g. licence needed
RRID: if applicable, e.g. RRID: SCR_014986

Note that this needs to be under an Open Source Initiative approved license where practicable compiled running software is made available. If the code is not hosted in a repository the GigaScience GitHub repository is also available for this purpose.

THIS SECTION IS NOW IN THE MANUSCRIPT, WITH THE NECESSARY INFORMATION PROVIDED

B) Availability of supporting data: (Add the following text)
"The protocol for this work is available in Protocols.io (add citation to references and cite reference number) [Ref#]. Further supporting data and snapshots of the code and visualization data are available in the GigaScience repository, GigaDB [Ref#].

WE HAVE INTRODUCED THIS SENTENCE IN THE MANUSCRIPT

C) List of abbreviations - please add all abbreviations used in the main text here in alphabetical order.

WE HAVE PROVIDED LIST OF ABBREVIATIONS

D) Funding: (after Competing interests)
All sources of funding for the research reported should be declared. The role of the funding body in the design of the study and collection, analysis, and interpretation of data and in writing the manuscript should be declared. Please use FundRef to report funding sources and include the award/grant number, and the name of the Principal Investigator of the grant.

WE HAVE PROVIDED SOURCE OF FUNDING

E) Authors' Contributions:
Please change the format to a table format, as per our Authorship Guidelines here:
https://academic.oup.com/gigascience/pages/authorship_guidelines

THIS IS NOW PROVIDED

F) References:
Please add the Protocols.io citation and renumber the references accordingly
Please add a place holder citation for the GigaDB DOI (Editorial will update this when ready)

THIS IS NOW ADDED

Their reports, together with any other comments, are below. Please also take a moment to check our website at https://giga.editorialmanager.com/ for any additional comments that were saved as attachments.

WE HAVE REVIEWED THE DATASET, AND IT IS COMPLETE

In addition, please register any new software application in the SciCrunch.org database to receive a RRID (Research Resource Identification Initiative ID) number, and include this in your manuscript. This will facilitate tracking, reproducibility and re-use of your tool.

WE HAVE DONE THIS

Close
