# Supplementary material for: Modeling genotypes in their microenvironment to predict single- and multi-cellular behavior
Source: Gigascience. 2019 Jan 31;8(3):giz010. doi: 10.1093/gigascience/giz010 (PMC6423375; doi:10.1093/gigascience/giz010)
Supplement: Reviewer_1_Report_Original_Submission -- Hidetoshi Mori — 7/31/2018 Reviewed [file giz010_reviewer_1_report_original_submission.pdf]

Reviewer Report

Title: Modelling genotypes in their microenvironment to predict single- and multi-cellular behaviour

Version: Original Submission **Date:** 7/31/2018

Reviewer name: Hidetoshi Mori

Reviewer Comments to Author:

It is not clear at all how authors prepared samples (dataset or algorithm) to analyze. They wrote "Specifically, we consider a population of homogenous pre-cancerous cells growing in a 3D environment; then, we gradually introduce mutations in onco- and tumour suppressor genes (Figure 1A) to study how they affect the multi-cellular growth (Figure 1B)." And Materials&Methods show "Cells as computational agents: Cells are represented in microC as computational agents with a physical dimension. Cells may interact with the microenvironment by consuming oxygen, and with other cells, via diffusible substances, such as cytokines, chemokines, growth factors, and hormones, whose production is defined by the network dynamics. This can be customised by the user. Furthermore, cells are also meta-agents, namely each cell is itself populated by a community of computational agents, the genes and molecules, which act and interact as a network inside the cell agent." These do not indicate how authors prepared each parameter. Were original biological models from cell lines or cells from genetically modified mouse mammary tumor or primary cells from patients? And what is this "physical dimension" and "physical (micro) environment? Physical (micro)environment usually indicates mechanical force from microenvironment, which is not well described and discussed in this manuscript. I guess that authors want to refer "tumor microenvironment" and its each content (cell-ECM, cell-cell, chemical and physical property) as their "dimensions". If so, this "physical dimension" and "physical (micro)environment" are not proper definition in this study.

WT sample indicated on Figure2C shows mass of cells, and the other indicated on Figure 4B shows scattered single cells without cell-cell association (epithelial polarization). Why these showed totally different predictions for 3D morphology? Can this predict normal tissue development? If so, this WT (with no mutation) should show normal epithelial morphology which is known as "acini" or ductal tubules. In current manuscript, there is no "normal" tissue compared. Therefore, it is not clear if this prediction is working fine or not. If this "WT" indicates a tumor with no mutation, then what kind of microenvironment induce these scattered (with less growth) or mass of cells?

P53 mutations are variable. Can this system analyze the effect of mutant p53 functions and compare WT and p53-null conditions? And then what is the difference between these?

Cancer progression include invasive conversion. Can this program predict invasive phenotype in 3D system? In current manuscript, it seems that only cell growth and cell death/necrosis are highlighted. Therefore, the invasiveness might have been ignored in this work.

"Hormone" is used as a parameter in this study. However, signaling from hormone is depending on the levels of hormone and hormone receptor. Are these parameters correctly used in this system? So far, hormone diffusion is only taken in account, meaning that the authors only consider the condition in the presence of hormone. Can the prediction also compare with or without hormone and hormone

receptor?
This may be the most important point to understand the significance of this study. If the actual biological 3D culture assay with these cancer cells with different levels of EGFR, PTEN and P53 is performed, does the predicted 3D morphology (and its growth pattern of each clone) in this simulation match to an actual 3D cell culture result? And the difference between actual 3D growth of cancer cells and predicted 3D morphology should be discussed well in this manuscript. At least, 'real' 3D culture result should be tested and compared.

Methods

Are the methods appropriate to the aims of the study, are they well described, and are necessary controls included? Choose an item.

Conclusions

Are the conclusions adequately supported by the data shown? Choose an item.

Reporting Standards

Does the manuscript adhere to the journal's guidelines on minimum standards of reporting? Choose an item.

Choose an item.

Statistics

Are you able to assess all statistics in the manuscript, including the appropriateness of statistical tests used? Choose an item.

Quality of Written English

Please indicate the quality of language in the manuscript: Choose an item.

Declaration of Competing Interests

Please complete a declaration of competing interests, considering the following questions:

- Have you in the past five years received reimbursements, fees, funding, or salary from an organisation that may in any way gain or lose financially from the publication of this manuscript, either now or in the future?
- Do you hold any stocks or shares in an organisation that may in any way gain or lose financially from the publication of this manuscript, either now or in the future?
- Do you hold or are you currently applying for any patents relating to the content of the manuscript?

- Have you received reimbursements, fees, funding, or salary from an organization that holds or has applied for patents relating to the content of the manuscript?
- Do you have any other financial competing interests?
- Do you have any non-financial competing interests in relation to this paper?

If you can answer no to all of the above, write 'I declare that I have no competing interests' below. If your reply is yes to any, please give details below.

I declare that I have no competing interests

I agree to the open peer review policy of the journal. I understand that my name will be included on my report to the authors and, if the manuscript is accepted for publication, my named report including any attachments I upload will be posted on the website along with the authors' responses. I agree for my report to be made available under an Open Access Creative Commons CC-BY license (http://creativecommons.org/licenses/by/4.0/). I understand that any comments which I do not wish to be included in my named report can be included as confidential comments to the editors, which will not be published.

I agree to the open peer review policy of the journal

To further support our reviewers, we have joined with Publons, where you can gain additional credit to further highlight your hard work (see: https://publons.com/journal/530/gigascience). On publication of this paper, your review will be automatically added to Publons, you can then choose whether or not to claim your Publons credit. I understand this statement.

Yes Choose an item.
