# Supplementary material for: Modeling genotypes in their microenvironment to predict single- and multi-cellular behavior
Source: Gigascience. 2019 Jan 31;8(3):giz010. doi: 10.1093/gigascience/giz010 (PMC6423375; doi:10.1093/gigascience/giz010)
Supplement: Reviewer_2_Report_Original_Submission -- Colin Paul — 8/5/2018 Reviewed [file giz010_reviewer_2_report_original_submission.pdf]

Reviewer Report

Title: Modelling genotypes in their microenvironment to predict single- and multi-cellular behaviour

Version: Original Submission **Date:** 8/5/2018

Reviewer name: Colin Paul

Reviewer Comments to Author:

In the manuscript "Modeling genotypes in their physical microenvironment to predict single- and multi-cellular behavior," Voukantsis and colleagues present an open-source, agent based modeling framework for modeling the behavior of cells in three dimensional environments. Agent decisions (at the cellular level) are based on activation status of prescribed gene networks, which drive cellular behaviors, namely proliferation, necrosis, apoptosis, or quiescence. These behaviors lead to emergent and time-dependent properties that are monitored.

The ability to model and interrogate cell behavior at the single-cell level and incorporate mutations and heterogeneity in the model will make it a useful tool for understanding cell proliferation in three dimensions. As the authors note, using the model in combination with single-cell imaging and sequencing methods could provide important insights into how underlying genetic/cell signaling activity influence cell phenotype. The reviewer envisions that the model, in its current implementation, will be most useful to experimentalists studying cell spheroid growth in various three dimensional cell culture matrices and for drug discovery applications aimed at inhibiting primary tumor growth or stem cell survival/expansion.

Due to some limitations in the modeling of the physical microenvironment in the current implementation of the model, the reviewer suggests minor revisions, primarily in language used to describe the model, prior to publication.

Major comments:

1) The authors emphasize that the model can "account for the effect of the physical microenvironment." While certain aspects of the physical environment are accounted for, including the location of the cells in three dimensions, the presence of neighboring cells, and local chemical concentraitons, other aspects are not included. Notably, physical factors such as matrix porosity, matrix stiffness, and topographical cues are not included. These physical cues are important in cell behavior (see, for example, Haeger et al., Biochimica et Biophysica Acta, 1840(8):2386-2395 (2014)). Attempts have recently been made to model some aspects of these physical cues (Ahmadzadeh et al., PNAS 114(9):E1617-1626 (2017)). While the absence of these physical cues in the manuscript does not invalidate the model, the physical aspects that are included should be clarified, and caveats about what is not modeled should be made. Instead of stating that the model accounts for the physical microenvironment, the authors should state that the model accounts for certain important aspects of the physical microenvironment. This could look similar to the second statement in the "Cells as computational agents" section.

2) On a similar note, one particular cell behavior that is particularly important to cancer biology, namely cell motility, is not included as a potential cell behavior in the model. Particularly in the case of cell spheroid growth, the "go or grow" dichotomy that dictates whether cells in a spheroid divide or migrate

out of the spheroid is of interest. See, for example, Garay et al., Experimental Cell Research 319(20):3094-3103 (2013) and Jimenez et al., Oncotarget 6(41):43438-43451 (2015). The authors should make it clear that cell migration was not considered in the model and indicate situations where this assumption is most appropriate.

3) Page 6, line 1 - "Cell-fate decisions are considered to be terminal, namely once they are triggered the network simulation fo the gene network stops." Does this mean that cells that enter a profliferative state, for example, proliferate indefinitely? Or are there multiple simulations of the gene network at each time step before a cell fate decision is made? Please clarify here. It appears that this issue is addressed in part in section 3 of the results, but it would be helpful to emphasize it here.

Minor comments:

1) The supplementary user guide and supplementary figures are nicely done. In particular, the sensitivity analyses in the supplementary figures are very informative and are presented well.

2) Page 8, line 19 - typo in "framework"

3) Page 9, line 9 - should state "three possible decisions"

4) Page 13, lines 1-9 is a nice example of how changing environmental cues can effect cell phenotype and tumor heterogeneity.

5) Page 13, line 27 - "section 4" should read "section 5."

6) Page 14, line 36 - which other networks besides the MAPK network does microC come with? What applications would these other networks be useful for?

Methods

Are the methods appropriate to the aims of the study, are they well described, and are necessary controls included? Choose an item.

Conclusions

Are the conclusions adequately supported by the data shown? Choose an item.

Reporting Standards

Does the manuscript adhere to the journal's guidelines on minimum standards of reporting? Choose an item.

Choose an item.

Statistics

Are you able to assess all statistics in the manuscript, including the appropriateness of statistical tests used? Choose an item.

Quality of Written English

Please indicate the quality of language in the manuscript: Choose an item.

Declaration of Competing Interests

Please complete a declaration of competing interests, considering the following questions:

- Have you in the past five years received reimbursements, fees, funding, or salary from an organisation that may in any way gain or lose financially from the publication of this manuscript, either now or in the future?
- Do you hold any stocks or shares in an organisation that may in any way gain or lose financially from the publication of this manuscript, either now or in the future?
- Do you hold or are you currently applying for any patents relating to the content of the manuscript?
- Have you received reimbursements, fees, funding, or salary from an organization that holds or has applied for patents relating to the content of the manuscript?
- Do you have any other financial competing interests?
- Do you have any non-financial competing interests in relation to this paper?

If you can answer no to all of the above, write 'I declare that I have no competing interests' below. If your reply is yes to any, please give details below.

I declare that I have no competing interests.

I agree to the open peer review policy of the journal. I understand that my name will be included on my report to the authors and, if the manuscript is accepted for publication, my named report including any attachments I upload will be posted on the website along with the authors' responses. I agree for my report to be made available under an Open Access Creative Commons CC-BY license (http://creativecommons.org/licenses/by/4.0/). I understand that any comments which I do not wish to be included in my named report can be included as confidential comments to the editors, which will not be published.

I agree to the open peer review policy of the journal

To further support our reviewers, we have joined with Publons, where you can gain additional credit to further highlight your hard work (see: https://publons.com/journal/530/gigascience). On publication of this paper, your review will be automatically added to Publons, you can then choose whether or not to claim your Publons credit. I understand this statement.

Yes Choose an item.
